# A high spatial resolution transcriptomics of tension wood reveals the genetic program of contractile G-layer formation

**DOI:** 10.3389/fpls.2026.1929884

**Published:** 2026-09-23

**Authors:** Manoj Kumar, Carolin Grones, Holly Allen, Judith Lundberg-Felten, Nicolas Delhomme, Bjorn Sundberg, Ewa J. Mellerowicz

**Affiliations:** 1 Umeå Plant Science Centre, Department of Forest Genetics and Plant Physiology, Swedish University of Agricultural Sciences, Umeå, Sweden; 2 BioFrontiers Institute and Department of Molecular, Cellular & Developmental Biology, University of Colorado Boulder, Boulder, CO, United States

**Keywords:** contractile cell wall, gelatinous layer, *Populus*, tension wood, transcriptomics

## Abstract

Tension wood (TW) formation in *Populus* involves the differentiation of G-fibers that deposit a cellulose-rich, contractile gelatinous layer (G-layer). Despite extensive study, the biosynthetic programs underlying G-layer formation have remained incompletely resolved, in part due to imprecise sampling of developmental stages. Here, we generate a high spatial-resolution transcriptome atlas of developing *Populus tremula* wood by tangential cryo-sectioning of TW and normal wood (NW) forming tissue. We overlaid microscopically determined cell wall thickening profiles with whole-transcriptome data, resulting in seven distinguishable TW and three NW stages and a clear separation of early and late G-layer-specific transcriptional programs during TW formation. Across 22,363 expressed genes, 90 co-expression clusters captured major programs defining cell wall biosynthesis, signaling, cytoskeleton dynamics, and stress responses. Differential expression analysis identified 2,898 genes specifically regulated during early or late G-layer formation—indicating a massive transcriptional reorganization unique to TW. Genes involved in cellulose, rhamnogalacturonan I, arabinogalactan II, and callose metabolism showed distinct induction patterns, including CAZymes not previously implicated in G-layer formation. We also identified G-layer-specific genes, including *XTH26* (*PtXTH5*), *SCL18*, *AGL29*, and genes encoding FLA12-like AGPs and LIM protein. Thirty transcription factors different than the secondary wall-inducing *NACs* and *MYBs*, and several genes involved in hormonal regulation specifically induced at the onset of G-layer formation represent novel putative regulators of the tertiary G-layer differentiation. Together, this spatially resolved transcriptome provides the most detailed map to date of the molecular program underlying TW formation, offering new insights into G-layer biosynthesis, signaling networks, and cell wall specialization in woody plants.

## 1 Introduction

Contractile cell walls, known as gelatinous cell wall layers (G-layers), are widespread in terrestrial plants (Gorshkova et al., 2018). Their main function is to generate tension within the growing organs, which can lead to organ bending as in the case of tension wood (TW), which bends stems with secondary growth; uplifting, for example, to maintain branch angle; twining, in the case of tendrils, to add support to vines; reinforcing plant anchorage in the soil, as in the case of contractile roots; burying organs in the soil, as in the case of contractile storage roots; and organ reinforcing, as, for example, in shaken stems. Mechanical properties of contractile walls fascinated generations of researchers (Clair et al., 2008; Mellerowicz and Gorshkova, 2012; Fournier et al., 2014; Alméras and Clair, 2016). Mechanically, G-layers can be described as exceptionally stiff and strong in tension, highly anisotropic, moisture-sensitive, and viscoelastic, making them an attractive compound for nanocellulose production used for example as synthetic fibers and cellulose-based tension actuators (De France et al., 2020). Among different models to study G-layers, primary phloem fibers in herbaceous plants and secondary TW tissues in hardwoods emerged as the most studied examples (Sousa-Baena and Onyenedum, 2022).

TW is a specialized form of wood found in hardwood species that generates high axial tension in the stems (Mellerowicz and Gorshkova, 2012; Fagerstedt et al., 2014). It can be induced by altering the position of stems in gravitational field. Typically, it forms at the upper side of bent stems. Its formation is characterized by an accelerated cambial growth, promotion of fiber differentiation at the expense of tracheary element differentiation, and formation of specialized tertiary G-layer in TW fibers (G-fibers) that is not formed in normal wood (NW) fibers. The G-layer is strikingly different from secondary wall layers (S-layers) formed before. It has a much higher content of highly crystalline cellulose, its cellulose microfibrils have larger diameters and more axial orientation (microfibril angle less than 10°), no or almost no lignin, and specialized composition of matrix polysaccharides.

Utilizing TW of hardwoods to study contractile walls provides several advantages over primary phloem fibers: (i) TW formation can be induced in a simple and efficient manner by tilting stems undergoing secondary growth, (ii) this approach yields increased amounts of G-layer-containing tissue, (iii) tissue isolation is relatively straightforward because TW is confined to the easily accessible upper side of the stem, and (iv) specific developmental stages of G-layer formation can be selected based on the distance of the developing tissue from the vascular cambium (Bygdell et al., 2017). This enables the collection of defined developmental stages of the cell wall deposition by tangential sectioning of developing wood.

Several omics approaches were devised to capture changes in gene expression needed for initiation and synthesis of G-layers including contrasting TW with NW or opposite wood formed on the lower side of inclined hardwood stems (Andersson-Gunnerås et al., 2006; Liu et al., 2021), the time-course analyses from the beginning of stem tilting (Paux et al., 2005; Cai et al., 2018; Zinkgraf et al., 2018), and most recently, the single-cell transcriptomics of a few cell layers of developing tension, normal, and opposite wood (Hsieh et al., 2025). However, achieving high-resolution transcriptomic coverage across all developmental stages has remained challenging, as advanced cell wall maturation and programmed cell death impede protoplast isolation and thus limit access to later stages of cellular development.

Here, we used tangential sectioning of developing TW and NW to obtain high-resolution spatially resolved transcriptomic information on genes induced at all developmental stages of G-layer formation. This approach, although lacking the cell-type resolution possible with single-cell transcriptomic technology, is particularly suitable to study cell wall dynamic changes during G-fiber differentiation, because these fibers constitute the bulk of TW. Thus, the current high-resolution sampling across NW and TW developmental zones provides highly precise information on gene expression during successive stages of G-layer deposition, identifying, among others, specific cell wall-related genes, transcription factors (TFs), and genes involved in carbon flux and hormonal regulation, which are induced or regulated during G-layer formation. The data provide solid information for functional analyses of TW formation and the possibility of biotechnological deployment of this unusual type of biomass.

## 2 Results and discussion

### 2.1 High-resolution sampling across tension wood-forming tissues

TW formation in *Populus tremula* was analyzed in two trees (denoted t08 and t11) bent for 3 weeks during the earlywood formation, whereas NW formation was followed in two upright trees (denoted n05 and n08). Both TW trees showed a wide arc of increased growth with typical G-fibers at the upper side of the bent stem. High spatial resolution sampling for RNA sequencing was achieved by sequential tangential cryo-sectioning across TW and NW developmental zones. Cell wall thickening was used as a measure of cell wall development. Assuming a constant rate of cell production, the slope of thickening curves (**Figure 1**) will reflect the rate of cell wall thickening. The cell wall thickness of the upright trees increased uniformly up to a radial distance of approximately 270 µm and then increased more slowly until approx. 500 µm (**Figure 1**), and the tangential sections from these trees were pooled into three samples: two samples covering the main cell wall growth phase and the third sample covering the final growth phase (**Supplementary Table S1**). In TW-forming trees, the cell wall thickness increased across a longer distance (ca. 700 µm), and a sharp increase in cell wall thickening rate corresponding to G-layer biosynthesis (Clair et al., 2011) was observed approximately 220 and 250 µm from the reference point (beginning of secondary wall deposition) in t11 and t08, respectively. A similar radial shift between t11 and t08 was observed for the end of cell wall thickening which occurred at 600 and 690 µm from the reference point, respectively, indicating that the development of the cell wall was accelerated in t11 compared to t08. Based on these measurements, tangential sections from the TW forming trees were pooled into six samples to capture all stages of cell wall biosynthesis (**Supplementary Table S1**).

**Figure 1:**
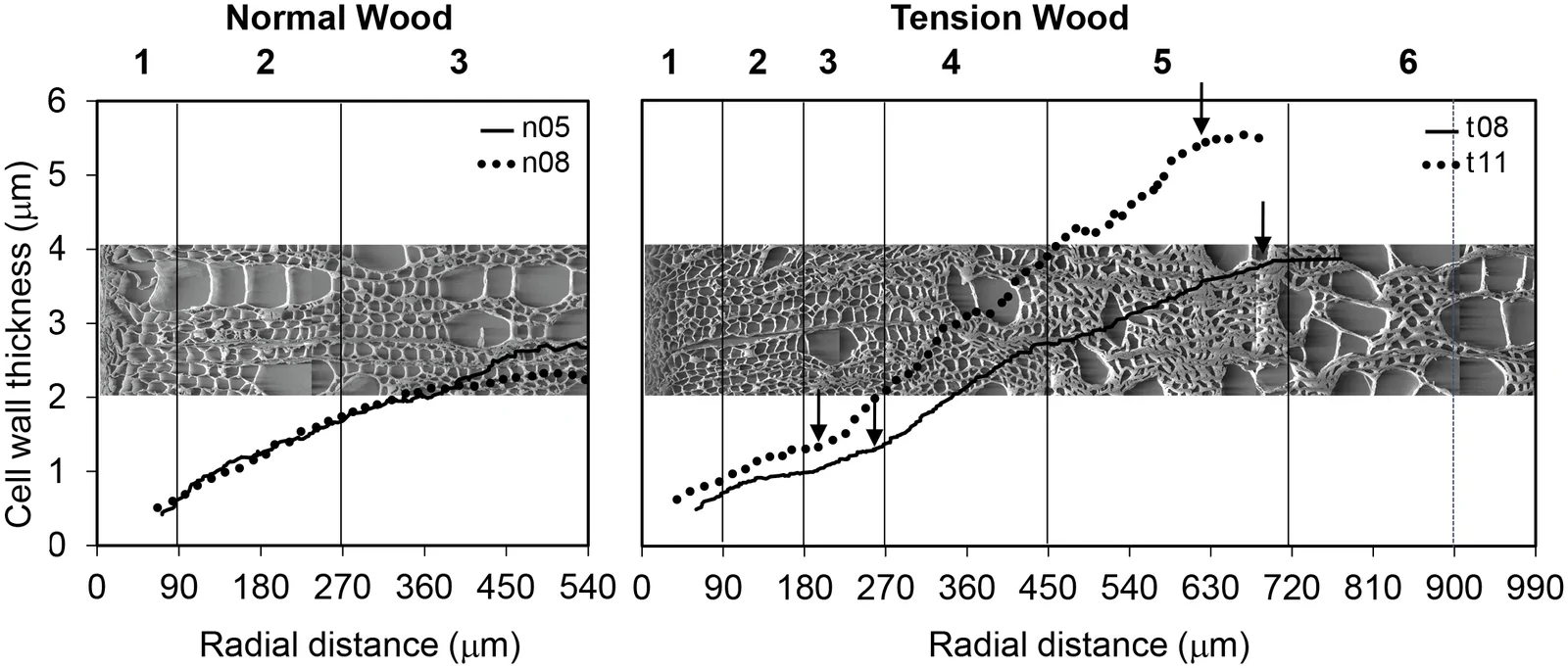
Cell wall thickening profiles and the approximate localization of samples for RNA analysis collected across developing normal wood (NW) and tension wood (TW). Samples were collected by pooling 30 μm tangential cryo-sections from two trees forming NW (n05 and n08) and two trees forming TW (t08 and t11), as shown in **Supplementary Table S1**. Cell wall thickening profiles were obtained from SEM images of wood collected immediately above the cryo-sectioned sample block. The wall thickness (from lumen to lumen) was measured for consecutive fiber cells in 10 radial files. The graphs represent the moving average (± 40). Radial distance was measured from the reference position. Arrows indicate the beginning and the end of the thickening of the gelatinous layer (G-layer) in TW forming trees. Approximate position of samples along the developmental gradient is indicated by numbers 1–3 for NW and 1–6 for TW. Note that cell wall development is accelerated in t11 compared to t08, as evidenced by the radial shift of the beginning and the end of the G-layer thickening.

### 2.2 Early transcriptional similarity and stage-specific reprogramming distinguish tension wood from normal wood development

We next assessed transcriptome dynamics during NW and TW formation. Principal component analysis (PCA) of all samples (22,363 expressed genes; **Supplementary Table S2**) showed that component 1 and component 2 explained 40% and 16% of the total variance, respectively (**Figure 2A**). Biological replicates of NW consistently clustered together, while replicates of TW showed a shift, with t11 samples grouping with t08 samples collected slightly further from the cambium, in agreement with the morphological shift observed between replicate trees (**Figure 1**). To account for this variation, we regrouped the samples into developmentally synchronized stages N1–N3 for NW and T1–T7 for TW (**Figure 2A**). Separation between NW and TW samples started from the third sampled stage (T3, N3), indicating that transcriptome of TW during the first two developmental stages resembles that of NW.

**Figure 2:**
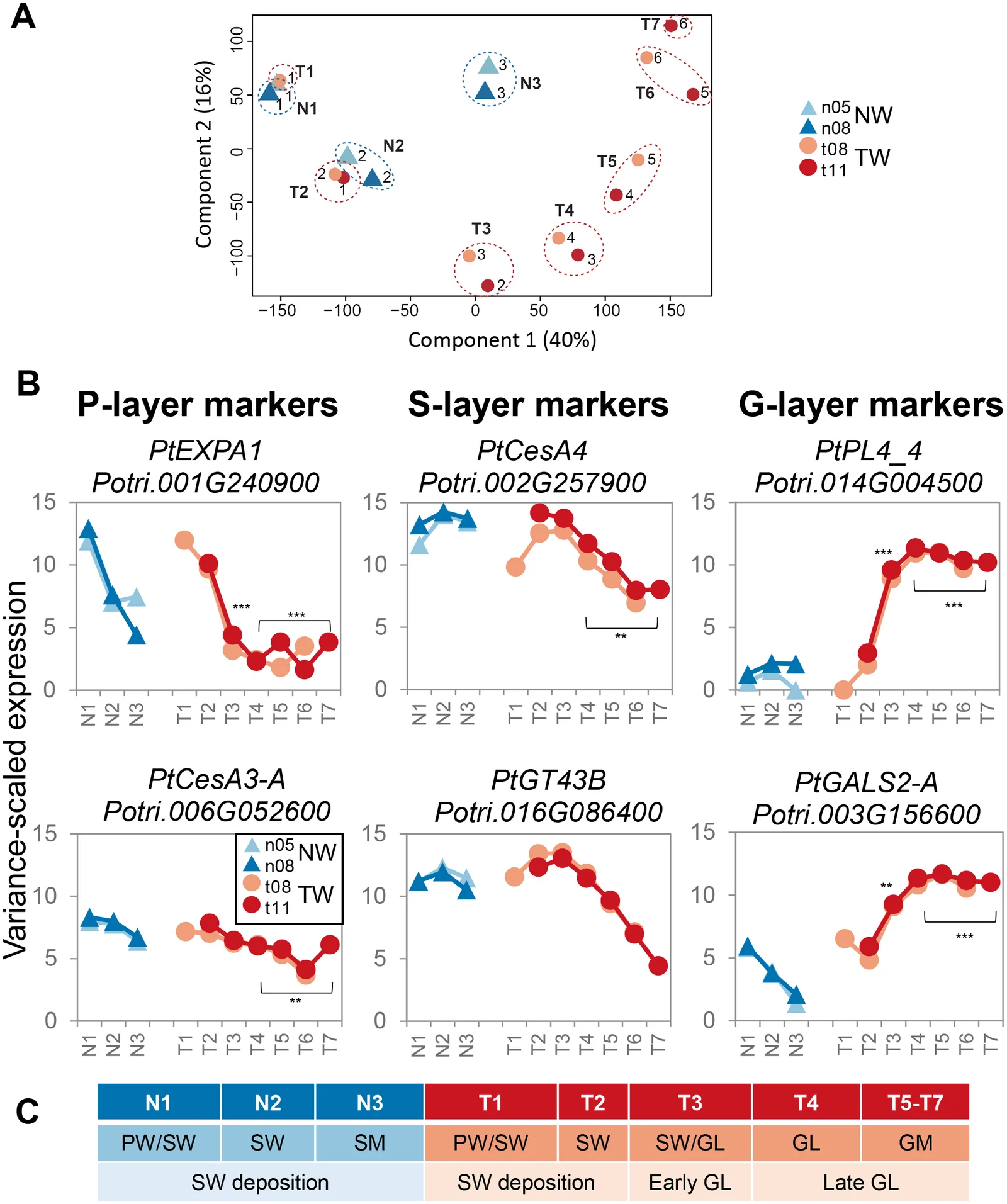
Definition of different stages of cell wall formation in the samples collected from different trees. **(A)** Principal component (PCA) analysis showing the grouping of the samples along the developmental gradient in normal (NW) and tension wood (TW). Based on similarity in gene expression, the samples were grouped into wood developmental stages as indicated by the ovals: N1–N3 for NW and T1–T7 for TW. **(B)** Expression of cell wall layer markers in the samples aligned according to developmental stages. Data are shown for two trees (n05 and n08) for NW and (t08 and t11) for TW. Stars indicate differentially expressed genes during early (T3) or late (T4–T7) G-layer formation compared to the mean expression at T1 and T2; **p ≤ 0.01, ***p ≤ 0.001, not significant (*p* > 0.05) when not shown. **(C)** Schematic representation of the deduced stages of cell wall formation in NW and TW, based on the transcriptome, expression of marker genes, and cell wall thickening. PW/SW, primary to secondary wall transition; SW, rapid secondary wall deposition; SM, secondary wall deposition and maturation; GL, rapid G-layer deposition; GM, G-layer deposition and maturation.

To refine the stages of NW and TW development, we followed the expression profiles of genes with known function during primary wall (P-layer), secondary wall (S-layer), and G-layer biosynthesis. The wood cell expansion marker, α-expansin *PtEXPA1* (Sundell et al., 2017), and the primary wall cellulose synthase, *PtCesA3-A* (Kumar et al., 2009), served as markers for primary cell wall development (**Figure 2B**). Both genes showed decreasing expression along the NW and TW developmental trajectory, although with different dynamics, the expression of *PtCesA3-A* decreasing much more gradually. To pinpoint the onset of S-layer formation, we analyzed the expression of the secondary wall CesA *PtCesA4* (Kumar et al., 2009) and the xylan biosynthetic gene *PtGT43B* (Ratke et al., 2015) (**Figure 2B**). The expression of both markers was already high at the beginning of the developmental series, peaked at stages N2 and T2–T3, and was then suppressed. These profiles indicate that the first samples collected in each series correspond to primary/secondary wall transition that follows cell expansion, with the main S-layer biosynthesis phase occurring at N2 and T2. After T3, the expression of both S-layer markers was suppressed in TW series, indicating the transition to G-layer formation. This assumption was confirmed by the sharp increase of the G-layer markers, rhamnogalacturonan lyase *PtPL4_4* and β-1,4-galactan synthase encoding gene, *PtGALS2-A* (Andersson-Gunnerås et al., 2006), which are likely directly involved in G-layer biosynthesis (Gorshkova et al., 2015; Guedes et al., 2017). *PtPL4_4* showed no expression in NW, so it is a specific marker of G-layer biosynthesis, whereas *PtGALS2-A* was also expressed at the early stage of NW formation, suggesting that it also functions in primary wall biosynthesis. High levels of the G-layer markers throughout the subsequent stages of TW development indicate that the biosynthetic machinery for G-layer formation remains active throughout later stages of TW differentiation. The expression profiles of marker genes confirmed the developmental offset between the two TW replicates, with the t08 series showing a developmental delay relative to t11.

Based on the cell wall thickening pattern (**Figure 1**), PCA analysis (**Figure 2A**), and the marker gene expression profiles (**Figure 2B**), we categorized the samples in the following way: N1–N3—NW S-layer biosynthesis, peaking at N2 and ending at N3 when axial system cells are expected to undergo programmed cell death; T1–T2—TW S-layer biosynthesis, peaking at T2; T3—S/G-layer transition denoted “early G-layer formation”; and T4–T7—G-layer biosynthesis, denoted “late G-layer biosynthesis”, with the main deposition at T4 (**Figure 2C**).

### 2.3 Identification of differentially regulated genes during G-layer biosynthesis

To focus on the genes uniquely induced or repressed during early and late G-layer formation, we carried out differential gene expression analyses setting a high stringency (pAdj ≤ 0.01 and |log2 fold change| ≥ 1) and comparing expression at T3 with the mean expression at T1 and T2 [early G-layer differentially regulated genes (DRGs)] or the mean expression at T4 to T7 with the mean expression at T1 and T2 (late G-layer DRGs) (**Supplementary Table S3** and **S4**). We also compared the NW developmental stages N2 and N3 with N1 (**Supplementary Table S5**). Despite the low number of replicates used in our study (two trees) and high stringency of analysis, as many as 2,898 DRGs were found during early and late G-layer development, whereas only 58 DRGs were found during the main and late stage of S-layer development in NW (**Figure 3A**; **Supplementary Table S6**). This indicates that the TW developmental series encompasses the highly dynamic phase of cell wall formation, whereas the NW samples primarily represent stages of S-layer deposition. The high resolution in G-layer-associated transcriptomes sufficiently captures the major transcriptional reprogramming during TW formation, whereas transcriptional changes during S-layer deposition in NW proceeds with comparatively minor changes in gene expression.

**Figure 3:**
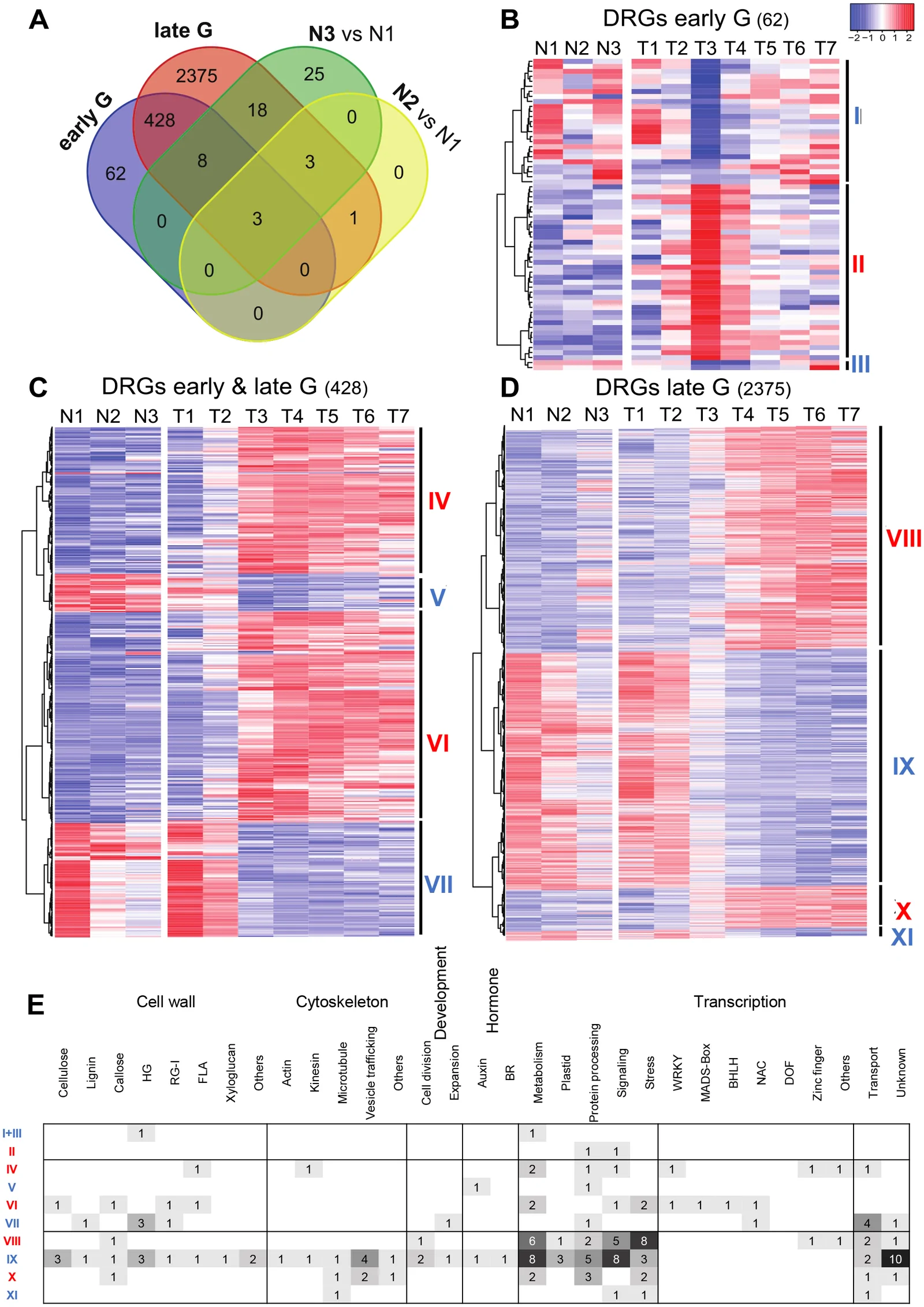
Differentially regulated genes during the onset and later development of the G-layer. **(A)** Venn diagram showing the distribution of differentially regulated genes (DRGs; pAdj |≤ 0.01; |log2 fold change|≥ 1) obtained from four transcriptome comparisons: N3 vs. N1, N2 vs. N1, early G (T3 vs. T1/T2), and late G (T4/T5/T6/T7 vs. T1/T2). **(B)** Heat map showing expression patterns (VST) of DRGs during the onset of G-layer formation (early G). The genes were clustered in three groups (I–III). **(C)** Heat map showing the expression patterns of genes differentially expressed in early and late G-layer formation stages. The genes were clustered into four groups (IV–VII). **(D)** Heat map of genes that were differentially expressed in late G stages. The genes were clustered into four groups (VIII–XI). **(E)** Enrichment of clusters I–XI in protein families (PFAMs) categorized according to their function. The number of PFAMs found in each group (I–XI) is shown.

DRGs were subsequently clustered into 11 co-expression groups (I–XI) based on their expression profile across the developmental gradient (**Figures 3B–D****;** **Supplementary Table S7**-**S9**) and their protein family (PFAM) enrichment analysis is shown in **Figure 3E** and **Supplementary Table S10**. Uniquely, early G-layer upregulated genes (group II) were enriched in signaling and protein processing, whereas downregulated ones (groups I and III) were enriched in metabolism. Groups upregulated both at the early and late stages of G-layer development (IV and VI) were enriched in genes involved in cellulose, callose, and rhamnogalacturonan I (RG-I) metabolism, encoding fasciclin-like arabinogalactan proteins (FLAs) and kinesins, and involved in transport, protein processing, signaling, and stress and in specific types of TFs, including WRKY, MADS, bHLH, and NAC. The downregulated groups (V and VII) were enriched in genes involved in lignification, pectin metabolism, cell expansion, auxin signaling, and protein processing and in the TFs of the DOF family. The groups upregulated uniquely during the late G-layer development (VIII and X) were enriched in genes related to callose metabolism, cell division, microtubule and vesicle trafficking, protein processing, signaling, stress, and zinc finger TFs. The downregulated groups (IX and XI) were enriched in genes related to metabolism of cell wall polysaccharides and lignin, cell division and expansion, auxin, and BR hormonal responses and were highly enriched in vesicle trafficking, metabolism, protein processing, signaling, and stress. Notably, group IX was most highly enriched in families of unknown function pointing to large gaps in our understanding of genes participating in late stages of G-layer formation.

### 2.4 Identification of genes involved in G-layer polysaccharide biosynthesis

To pinpoint genes directly involved in G-layer biosynthesis, we carried out CAZY gene annotation (**Supplementary Table S11**) and analyzed CAZymes and other known genes involved in biosynthesis of different cell wall polysaccharides. Out of 1,925 CAZyme genes (including expansins and GT116 family) identified in *Populus trichocarpa* V 3.0 assembly (Kumar et al., 2019; Amos et al., 2022), 890 genes were expressed in NW and TW samples, and the analysis of their expression across developmental series allows an unprecedented insight into the reprogramming of cell wall biosynthesis during TW differentiation.

#### 2.4.1 Reprogramming of CesA isoform usage and cellulose synthase complex-associated genes during G-layer formation

Cellulose content of the G-layer greatly exceeds that of P- or S-layers, and its structure exhibits unique properties such as higher crystallinity, thicker microfibrils, and low microfibril angle, which were proposed to play a key role in cell wall longitudinal contraction (Mellerowicz et al., 2008; Mellerowicz and Gorshkova, 2012; Gorshkova et al., 2018). In *Arabidopsis*, the cellulose of P- and S-layers is synthesized by plasma membrane localized primary and secondary cellulose synthase complexes, respectively, each composed of either primary wall CesAs (CesA1, 3, and 6) or secondary wall CesAs (CesA4, 7, and 8) in a 1:1:1 stoichiometry (Polko and Kieber, 2019). Poplar orthologs of these isoforms are denoted by capital letters (A, B, …F). *Pt*CesA4, 7-A, 7-B, 8-A, and 8-B (Kumar et al., 2009) are induced during S-layer formation (Sundell et al., 2017), but the stoichiometry among the corresponding proteins is unclear (Zhang et al., 2018). Moreover, both primary and secondary wall complexes probably participate in secondary cell wall formation (Xi et al., 2017). In the current study, secondary wall CesAs were co-expressed in the NW samples and in TW during S-layer formation, but their expression diverged during the G-layer formation with *PtCesA8-B* expressed at a higher level compared to other isoforms (**Figure 4A**), confirming previous results (Zhang et al., 2018). Moreover, many of the primary wall CesAs (e.g., *PtCesA1A*, *1B*, *3C*, *3D*, *6B, 6C*) were highly expressed throughout both NW and TW formation, and only some of them (e.g., *PtCesA 6A*, *6D*, *6E*) showed decreased expression at the late stages of G-layer formation (**Figures 4B–D**). These observations are reminiscent of the *CesA* gene expression reprogramming during G-layer differentiation in flax phloem fibers (Mokshina et al., 2017), and they jointly suggest that the primary wall CesA complexes might function not only during S- but also during G-layer biosynthesis.

**Figure 4:**
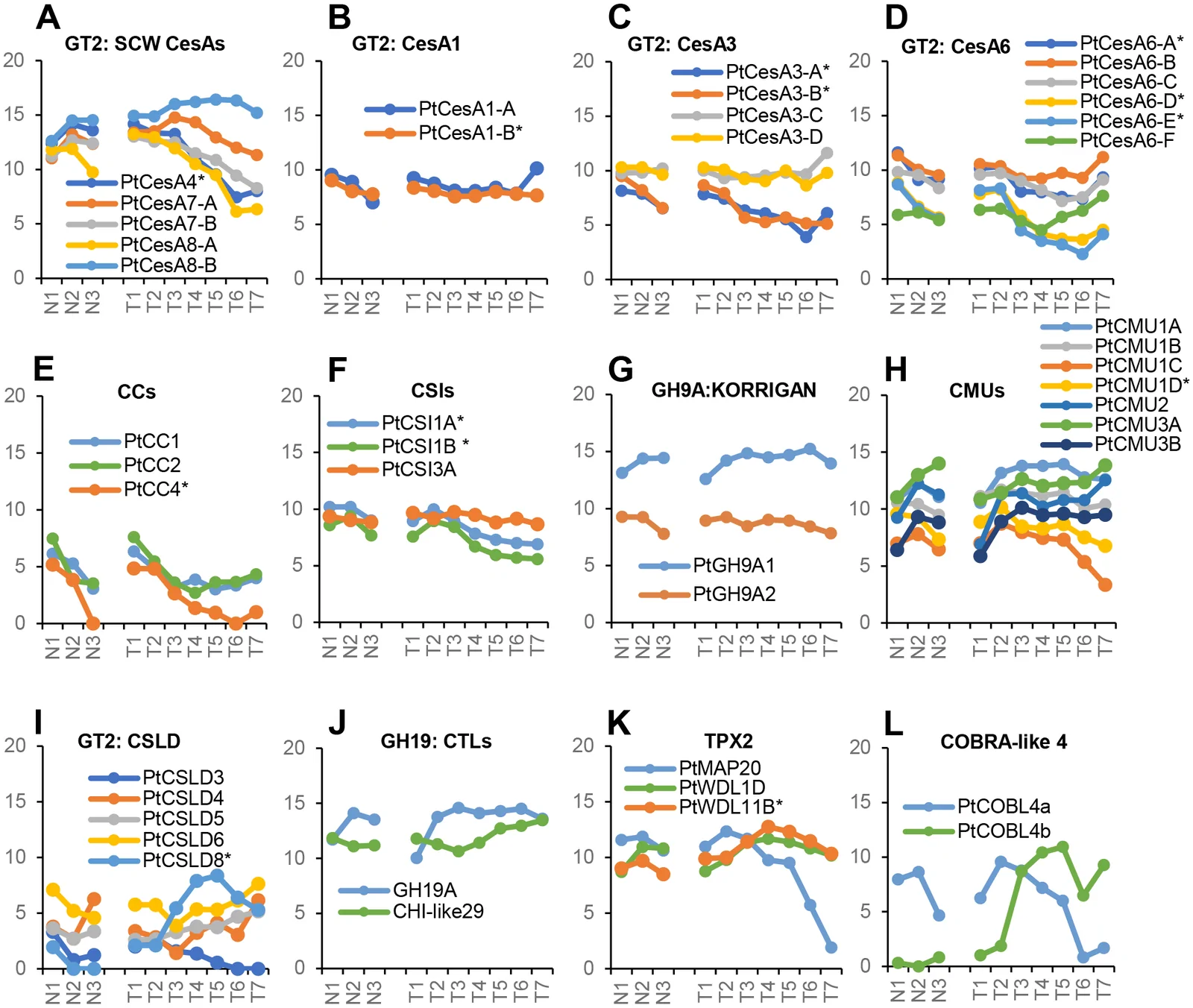
Expression of genes involved in cellulose biosynthesis. Average VST expression for two biological replicates (n05/n08 and t08/t11) is shown for groups of genes implicated in the biosynthesis of cellulose: secondary cell wall CesAs **(A)**, primary cell wall CesAs **(B–D)**, *COMPANION OF CELLULOSE SYNTHESIS* (*CC*) **(E)**, *CELLULOSE SYNTHASE INTERACTING* (*CSI*) **(F)**, *KORRIGAN* **(G)**, *CELLULOSE MICROTUBULE UNCOUPLING* (*CMU*) **(H)**, *CELLULOSE SYNTHASE LIKE D* (*CSLD*) **(I)**, *CHITINASE-LIKE* (*CTL*) **(J)**, genes encoding TPX2 domain microtubule-binding proteins **(K)**, and *COBRA-LIKE 4* (*COBL4*) **(L)**. Stars indicate differentially expressed genes during early or late G-layer formation. All CAZYmes IDs are listed in **Supplementary Table S11**. Gene IDs for other families: *PtCMU1A*—*Potri.003G143200*, *PtCMU1B*—*Potri.001G087800*, *PtCMU1C*—*Potri.002G173400*, *PtCMU1D*—*Potri.014G100400*, *PtCMU2*—*Potri.008G094700*, *PtCMU3A*—*Potri.015G075300*, *PtCMU3B*—*Potri.012G080100*, *PtCC1*—*Potri.014G026800*, *PtCC2*—*Potri.002G124600*, *PtCC4 - Potri.009G140600*, *PtCSI1A*—*Potri.007G087200*, *PtCSI1B*—*Potri.005G080100*, *PtCSI3A*—*Potri.005G180100*, *PtMAP20*—*Potri.T059900*, *PtWDL1D*—*Potri.010G076200*, *PtWDL11B*—*Potri.006G200400*, *PtCOBL4a*—*Potri.015G060100*, *PtCOBL4b*—*Potri.004G117200.*.

A few other proteins are part of the cellulose synthase complex, including COMPANION OF CELLULOSE SYNTHESIS (CC), CELLULOSE SYNTAHSE INTERACTING (CSI), KORRIGAN, and CELLULOSE MICROTUBULE UNCOUPLING (CMU) (Polko and Kieber, 2019). Expression of none of the corresponding genes was significantly increased during TW formation but was stable during the entire cell wall development in NW and TW, except for the *CC* genes, *PtCSI1A* and *B*, *PtCMU1C* and *D* that were suppressed during G-layer development (**Figures 4E–H**). This suggests that the structure of the cellulose synthase complex involved in G-layer synthesis might differ from the secondary wall cellulose synthase complex.

Cellulose synthase-like clade D (CSLD) proteins are GT2 family members closely related to CesAs, which have been implicated in cellulose biosynthesis in tip-growing cells (Yang et al., 2020). We found that one *CSLD* gene (*PtCSLD8/AtCSLD4*) was transiently induced and three others showed increasing expression during G-layer formation (**Figure 4I**). These genes might be involved in the synthesis of transversely oriented cellulose microfibrils in the tips of intrusively elongating fibers (Majda et al., 2021). Their increased expression in TW is consistent with increased elongation of TW compared to NW fibers (Jourez et al., 2001).

Among other putative contributors to cellulose biosynthesis, *CHITINASE-LIKE* genes from the GH19 family (*CTLs*) (Polko and Kieber, 2019) of *Populus*, *PtGH19A* and *PtCHI-like29*, were induced during early and late G-layer biosynthesis, respectively (**Figure 4J**), whereas the TPX2 domain-containing gene, *MICROTUBULE ASSOCIATED PROTEIN20* (*PtMAP20*) that is co-expressed with secondary wall *CesAs* in NW (Rajangam et al., 2008), was suppressed in TW (**Figure 4K**). The expression of other TPX2 genes, *PtWDL11B* and *PtWDL1D*, was, however, either upregulated or stable, respectively, during G-layer formation. *COBRA-LIKE4* (*COBL4*) required for cellulose biosynthesis in *Arabidopsis* (Xue et al., 2024) had two *Populus* homologs that displayed very different expression patterns; *PtCOBL4a* was downregulated, whereas *PtCOBL4b*/*PtCOB3* was *de novo*-induced during G-layer formation (**Figure 5L**), consistent with its essential role in the G-layer synthesis in poplar (Xu et al., 2024). Our dataset suggests that, although the overall S-layer cellulose biosynthesis machinery is not markedly altered during tension wood formation, successful G-layer formation may rely on the upregulation of a small subset of cellulose synthase complex-associated components (e.g., *Pt*CesA8-B, *Pt*CSLD8, *Pt*COBL4b/COB3, *Pt*WDL1D, *Pt*WDL11B, *Pt*CHI-like29). Further research is needed to investigate the role of these genes during TW formation.

**Figure 5:**
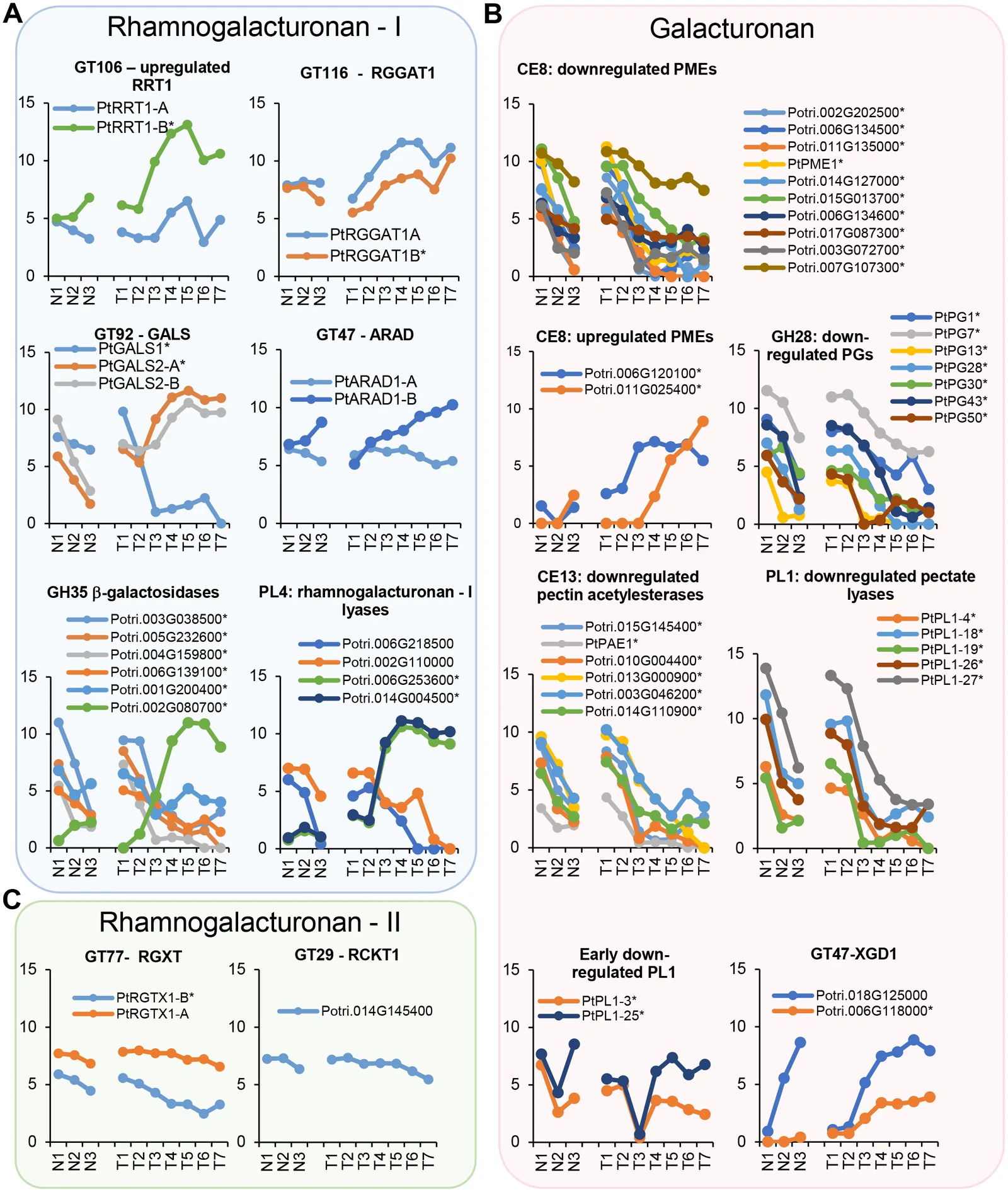
Pectin-related CAZY genes showing differential expression during G-layer biosynthesis. **(A)** Genes involved in rhamnogalacturonan I (RG-I) biosynthesis/modification encoding RG-I : Rhamnosyltransferases (RRTs) of GT106, RG-I : Galacturonosyltransferases (RGGATs) of GT116, galactan synthases (GALS) of GT92, arabinan : arabinosyltransferase (ARAD) of GT47, β-galactosidases of GH35, and RG-I lyases of PL4. **(B)** Genes involved in galacturonan biosynthesis/modification encoding: pectin methylesterases (PMEs) of CE8, polygalacturonases (PGs) of GH28, xylogalacturonan β-1,3-xylosyltransferase (XGD1) of GT47, pectate lyases of PL1, and pectin acetylesterases of CE13. **(C)** Genes involved in rhamnogalacturonan II (RG-II) biosynthesis: RG-II α-1,3-xylosyltransferase (RGXT) of GT77 and RG-II CMP-β-Kdo transferase (RCKT1) of GT29. Average VST expression. Stars indicate differentially regulated genes during the early or late phase of G-layer synthesis. Expression of all family members and *P. trichocarpa* gene IDs corresponding to the abbreviated names are listed in **Supplementary Table S11**.

#### 2.4.2 New candidate regulators of G-layer pectin biosynthesis and modification

In contrast to S-layers, the G-layer contains a substantial amount of pectins, of which RG-I is the most abundant (Mellerowicz and Gorshkova, 2012; Gorshkova et al., 2015, 2018). Its backbone is synthesized by RG-I : Rhamnosyltransferases (RRTs)] and RG-I : Galacturonosyltransferases (RGGATs), which have recently been characterized in *Arabidopsis* and other plants in GT106 and GT116 families, respectively (Takenaka et al., 2018; Wachananawat et al., 2020; Amos et al., 2022). We have identified homologs of the *AtRRT1-10* and *AtRGGAT1* genes in *Populus* and named them as in *Arabidopsis* genes adding capital letters for multiplied paralogs (**Supplementary Table S11**). *PtRRT1-A*, *PtRRT1-B*, *PtRGGAT1-A*, and *PtRGGAT1-B* exhibited G-layer-specific upregulation peaking at T5, in contrast to other *RRT* and clade A GT116 genes, and are therefore candidates for G-layer RG-I biosynthetic genes (**Figure 5A****;** **Supplementary Table S11**). RG-I galactan synthesized by RG-I galactan synthase (GALS; GT92) (Prabhakar et al., 2023) is the most abundant insoluble matrix polysaccharide of G-layer (Gorshkova et al., 2015), and *PtGALS2-A* is a G-layer marker. *Populus GALS* genes showed two diverged expression patterns during G-layer formation: downregulation (*PtGALS1*) or upregulation peaking at T5 (*PtGALS2-A*, *PtGALS2-B*) similar to G-layer RG-I backbone biosynthetic genes (**Figure 5A**). RG-I arabinan, synthesized by ARAD proteins from GT47 (Delmer et al., 2024), is also part of the G-layer structure (Gorshkova et al., 2015; Guedes et al., 2017). *Populus ARAD* genes showed constant expression *(ARAD1-A*) or mild upregulation *(ARAD1-B*) throughout G-layer deposition (**Figure 5A**).

The *in muro* trimming of the RG-I backbone and galactan side chains was proposed to play a key role in the generation of tension within G-layer cellulose macrofibrils by the entrapment mechanism in TW (Gorshkova et al., 2015; Mikshina et al., 2015). Moreover, in flax G-fibers, GH35 β-1,4-galactosidase *Lu*GAL1 was shown to be essential for maturation of the G-layer (Roach et al., 2011). Analysis of transcript abundance of *Populus* GH35 family members revealed that the majority were suppressed during S- and G-layer deposition, except for *Potri.002G080700*, which was transiently highly upregulated, peaking at T5 (**Figure 5A****;** **Supplementary Table S11**), making it a prime candidate for the previously observed G-layer β-1,4-galactosidase activity (Gorshkova et al., 2015). Two members of the rhamnogalacturonan lyase family (PL4) showed similar upregulation during G-layer biosynthesis (*Potri.006G253600* and *Potri.014G004500/PL4_4*—a G-layer marker), unlike other *PL4* genes (**Figure 5A**; **Supplementary Table S11**). *Potri.002G080700* is closely related to *Arabidopsis MUM2*/*BGAL6*, which is essential for the trimming of RG-I galactan and the hygroscopic swelling of mucilage in seeds (Macquet et al., 2007). Hygroscopic swelling of RG-I galactan inside cellulose macrofibrils could drive longitudinal contraction of G-layers (Fournier et al., 2014). Thus, *Potri.002G080700*, *Potri.006G253600*, and *Potri.014G004500/PL4_4* might be the key regulators of the G-layer contractile properties.

Homogalacturonan, which is the most abundant pectin of primary walls, was reported in the developing inner part of the G-layer (Bowling and Vaughn, 2008; Gritsch et al., 2015; Guedes et al., 2017). The polymer backbone of α-1,4-linked galaturonosyl residues is synthesized by GAUT and GAUT-like (GATL) proteins of family GT8 (Delmer et al., 2024), some of which also function in xylan reducing end sequence biosynthesis. We observed that homologs of pectin-related *GAUT* genes (*GAUT1–7*) were stably expressed in developing NW and TW and did not show differential regulation during G-layer development (**Supplementary Table S11**). Except *GALT1* and *GATL7* homologs, *Populus* GATL genes showed either a suppression (*PtGALT2A*) or a stable expression pattern in TW series (**Supplementary Table S11**). Altogether, these findings suggest no specific induction of galacturonan biosynthesis during G-layer formation. The primary walls of some tissues contain xylogalacturonan (XGA) substituted with β-1,3-Xyl*p* residues, which are added to the backbone by xylosyltransferase encoded by *XYLOGALACURONAN DEFFICIENT1* (*XGD1*) in family GT47 (Delmer et al., 2024). Two *Populus* copies of *XGD1* exhibited increasing expression during G-layer biosynthesis (**Figure 5B**), suggesting that this layer or G-fiber cell wall layers contain XGA. To our knowledge, there are no previous records on the presence of XGA in the G-layers, and this point should be addressed by future research. Homogalacturonan modifying enzymes encoding genes (*CE8*, *CE13*, *GH28*, and *PL1*) showed high expression levels during primary to secondary wall transition and strong suppression at later stages of NW and TW development (**Figure 5B**; **Supplementary Table S11**), which must be regulated. An exceptional expression pattern was observed for two PME genes (*Potri.006G120100* and *Potri.011G025400*), showing specific induction during G-layer biosynthesis, and two PL1 genes (*PtPL1-3* and *PtPL1-25*), showing a transient suppression at T3 (**Figure 6B**), suggesting their involvement in homogalacturonan modification in TW.

**Figure 6:**
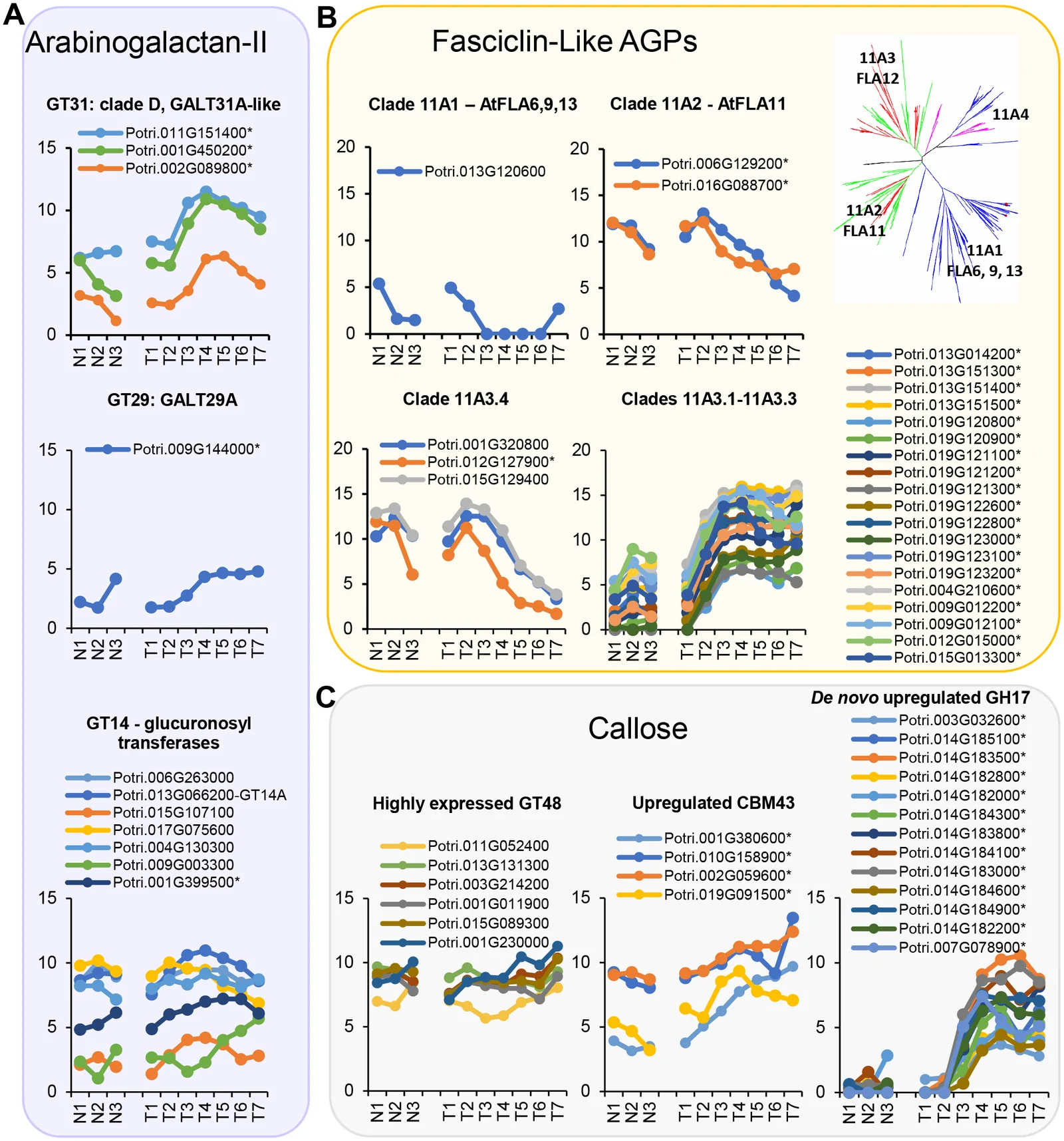
Expression of genes involved in the biosynthesis of arabinogalactan II **(A)**, fasciclin-like AGPs (FLAs, **B)**, and callose **(C)**. Average VST expression of selected genes discussed in the text is plotted, with the remaining genes shown in **Supplementary Table S11**, except for FLAs, where all selected clades (shown in the inset on the left) are included. Full phylogenetic analysis of FLAs is shown in **Supplementary Figure S1** and **Supplementary Table S12** and S13. CAZY gene families encoding β-1,6-galactosyl transferases (GALTs) from families GT31 and GT29, β-glucuronosyltransferases from GT14, callose synthases (GT48), callose hydrolases (GH17), and callose-binding proteins with CMB43 other than GH17. Stars indicate differentially expressed genes during the early and/or late phase of G-layer biosynthesis.

Rhamnogalacturonan II (RG-II) is an essential pectic polymer present in low amounts in primary cell walls. So far, there have been no reports on the presence of RG-II in G-layers. Two CAZY families have been implicated in the RG-II biosynthesis: GT77, which includes UDP-Xyl: RG-II α-1,3-xylosyltransferase with two characterized enzymes in *Arabidopsis*, RGXT1 and 2 (Delmer et al., 2024), and GT29 that has recently been proposed to include RG-II CMP-β-Kdo transferase RCKT1 (Zhang et al., 2024). *Populus* homologs were most highly expressed at the beginning of NW and TW developmental series and displayed a declining expression during TW differentiation (**Figure 5C**), suggesting that RG-II is limited to primary walls.

#### 2.4.3 Classification of a G-layer-specific FLA12 subclade

G-layers of flax and *Populus* contain large amounts of arabinogalactan II (AG-II), a water-soluble polymer known as glycosidic antennae of arabinogalactan proteins (AGPs) (Gurjanov et al., 2008; Gorshkova et al., 2015; Guedes et al., 2017). In aspen TW, an acidic AG-II component was identified as a polymer retained within cellulose macrofibrils along with RG-I galactan (Gorshkova et al., 2015). Early studies in beech TW suggested that pectic β-1-4, galactan and AG-II could be covalently connected (Meier, 1962). AG-II is synthesized by β-galactosyltransferases (GT29 and GT31), α-fucosyl transferases FUT4 and FUT6 (GT37), α-arabinosyl transferase RAY1 (GT77), and β-glucuronosyltransferases (GT14), which were characterized in *Arabidopsis* (Knoch et al., 2014). The corresponding *Populus* genes were identified (except for the fucosyl transferases) (Kumar et al., 2019), and majority showed upregulation during G-layer formation (**Figure 6A**), suggesting their involvement in G-layer biosynthesis.

The protein part of AG-II is likely a FLA, as the transcripts similar to *FLA12* are a hallmark of G-fiber biosynthesis both in TW and in the phloem (Lafarguette et al., 2004; Andersson-Gunnerås et al., 2006; Wang et al., 2015; 2017; Sousa-Baena and Onyenedum, 2022). The present analysis also found their significant enrichment among transcripts upregulated during G-layer biosynthesis (**Figure 3**). Since the automated annotation is not sensitive enough to correctly assign homology among different FLA proteins, we have carried out their extensive phylogenetic analysis (**Supplementary Table S12**, **S13****;** **Supplementary Figure S1**) to specify clades involved in TW formation. We distinguished five superclades (C11A–C11E), with C11A further subdivided into clades C11A1–C11A4. Remarkably, out of the 22 closest *FLA12* homologs in clade C11A3, 18 classified in the subclades C11A3.1–3.3 (**Supplementary Table S13**) showed significant upregulation during G-layer formation (**Figure 6B**). Majority of these genes are clustered on chromosomes 13 and 19. The remaining *FLA12* homologs formed a subclade C11A3.4 and were downregulated during G-layer formation as were also other *FLA* genes classified in clades C11A1 (containing *FLA6*, *9*, and *13*) and C11A2 (containing *FLA11*). In *Arabidopsis*, FLA12 (classified in subclade C11A3.5; **Supplementary Table S13**) increased cellulose content in secondary walls, whereas FLA11 stimulated lignin biosynthesis, which was solely determined by their AG-II antennae (Ma et al., 2022, 2023). These proposed functions are in line with the expression patterns in *Populus* (**Figure 6B**) with *FLA11* homologs (clade 11A2) coinciding with P- and S-layer lignification and majority of *FLA12* homologs (subclades 11A3.1–3.3) concurring with the biosynthesis of cellulose-enriched G-layers. Some of the G-layer-induced *FLA12* homologs appear to be G-layer-specific (e.g., *Potri.019G120800*, *Potri.019G121300*; *Potri.019G123000*), while others (e.g., *Potri.013G014200*; *Potri.013G151500*) are also expressed during S-layer deposition but at a lower level. Single-cell transcriptomic analysis (Hsieh et al., 2025) suggests that the induction of clade 11A3.1 *FLA12* genes in TW samples can vary among different cell types. The essential role of *FLA12* genes (subclades 11A3.1–3.3) in S- and G-layer formation is supported by functional studies in poplar (Wang et al., 2015, 2017).

#### 2.4.4 G-layer-specific induction of a chromosome 14 callose-metabolizing gene cluster

Information regarding the presence of callose in G-fibers is currently incomplete. Callose was immunolocalized in S-layer of G-fibers in vine coiling tendrils (Meloche et al., 2007), whereas callose plugs were detected in gravi-stimulated phloem but not xylem G-fibers of flax (Ibragimova et al., 2017). On the other hand, callose synthase transcripts were detected in TW of *Liriodendron tulipifera* (Jin et al., 2011). We have found enrichment in GH17 (callose hydrolase) and domain CBM43/X8 (callose-binding) genes among co-expressed DRGs during G-layer formation (**Figure 3**), prompting the expression analysis of callose-related CAZymes. We found two expression patterns in GH17 family: eight members were highly expressed at the primary/secondary wall transition and suppressed during G-layer biosynthesis, whereas 13 members were upregulated during G-layer biosynthesis with negligible or no expression in NW, thus representing *de novo*-induced genes (**Figure 6C****;** **Supplementary Table S11**). Majority (11) of them are clustered on chromosome 14 and do not have CBM43 or GPI domains (**Supplementary Table S14**).

The GT48 family members encoding callose synthases exhibited, in contrast, stable expression in NW and TW developmental series (**Supplementary Table S11**) with most highly expressed members shown in **Figure 6C**. Among other genes with CBM43, four showed upregulation during G-layer biosynthesis. These results indicate continuous callose biosynthesis at all developmental stages in NW and TW and a dedicated callose-metabolizing machinery during G-layer biosynthesis. Callose metabolism regulates symplasmic continuity via plasmodesmata or plugs, which is essential for gravitropic responses in primary growth (Han et al., 2014), but has not yet been studied in reaction wood.

#### 2.4.5 Expression of genes related to biosynthesis of hemicelluloses

Xyloglucan is essential for poplar stem righting and is thought to play a role in the generation of tension on the upper stem side (Mellerowicz et al., 2008; Baba et al., 2009). It was detected either as a matrix polymer associated with G-layers (Nishikubo et al., 2007; Baba et al., 2009; Hunt et al., 2026) or located between layers (S1/S2 and S2/G) (Bowling and Vaughn, 2008; Sandqvist et al., 2010; Guedes et al., 2017). Previous transcript analyses in TW detected upregulation of several XTH genes compared to either NW or opposite wood (Andersson-Gunnerås et al., 2006; Nishikubo et al., 2007; Chen et al., 2015; Gerttula et al., 2015), and a proteomic analysis identified *Pt*XTH6/XET16E as a G-layer-resident protein (Kaku et al., 2009). Moreover, bean *XTH5* gene was detected in developing G-layers of phloem G-fibers and was shown to respond to brassinosteroid (Hunt et al., 2026). However, there is a scarcity of information on the expression of xyloglucan biosynthetic genes during G-fiber differentiation. The core xyloglucan structure is synthesized by a Golgi protein complex consisting of a glucan synthase CSLC4 (GT2); three xylosyltransferases XXT1, 2, and 5 (GT34); two galactosyltransferases MUR3 and XLT2 (GT47); and a fucosyltransferase FUT1 (GT37) (Julian and Zabotina, 2022). Among the six *CSLC* genes expressed in developing wood, one (*PtCSLC4-B*) was upregulated and one (*PtCSL6-A*) was downregulated during G-layer biosynthesis, while the others were stably expressed in NW and TW, similar to all xyloglucan xylosyl transferases (**Figure 7A**; **Supplementary Table S11**). Moreover, among the expressed xyloglucan galactosyltransferases, *PtMUR3* was upregulated during late G-layer formation. These data indicate a maintained potential for xyloglucan synthase complex formation in TW, possibly with a dedicated glucan synthase (*Pt*CSLC4-B) and galactosyl transferase (*Pt*MUR3).

**Figure 7:**
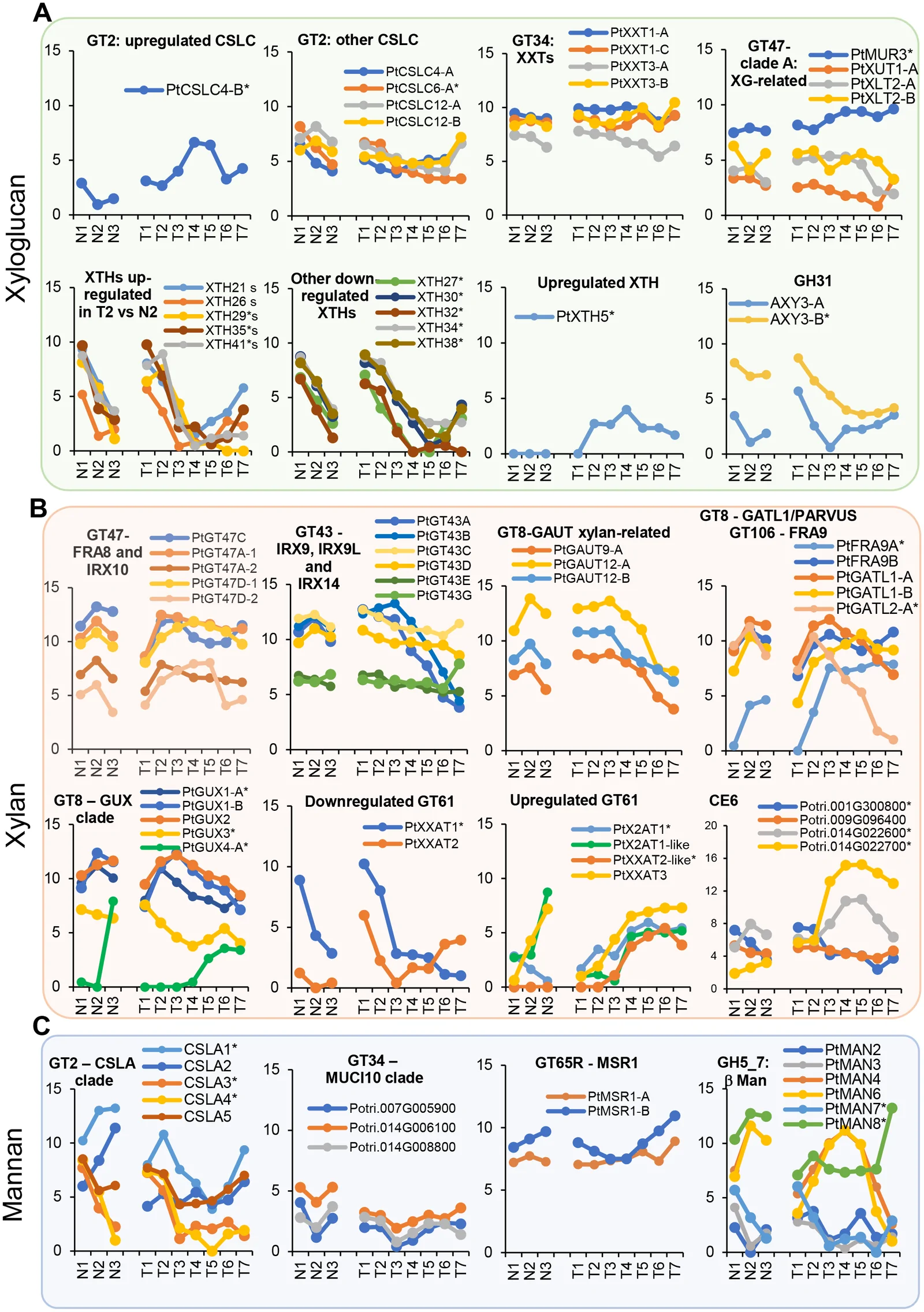
Expression of genes involved in the biosynthesis of hemicelluloses. **(A)** Genes involved in xyloglucan (XG) synthesis encoding XG synthases from GT2 (CSLCs), XG α-1,2-xylosyltransferase (XXTs) from GT34, galactosyltransferases from clade A of GT47, XG endo-transglucosylases/hydrolases (XTH) from family GH16, and upregulated α-xylosidases from family GH31. **(B)** Genes involved in xylan synthesis encoding xylan synthases from GT47, accessory proteins of xylan synthase from family GT43, reducing end synthesizing GTs from family GT8 (GAUT and GATL clades), GT47 (FRA8) and GT106 (FRA9), glucuronosyltransferases from the GUX clade of GT8, xylosyl- and arabinosyltransferases from family GT61, and xylan acetyles terases from family CE6. **(C)** Genes involved in mannan synthesis encoding mannan synthases from the CSLA clade of GT2, galactosyltransferases from the MUCI10 clade of family GT34, MSR1 homologs from family GT106, and endo-β-1,4 mannanases from clade 7 of family GH5. Average VST expression. Expression of remaining CAZy family members and Potri codes corresponding to the short names are shown in **Supplementary Table S11**. *Populus* enzymes were named after Kumar et al. (2019). Stars indicate differentially expressed genes during the early and/or late G-layer formation; “s” indicates significantly upregulated XTH genes during secondary wall formation in TW compared to NW (T2 compared to N2) based on post-ANOVA contrast analysis (*p* ≤ 0.05).

Xyloglucan endotransglucosylation in cell wall was proposed as an important reaction maintaining contact among different cell wall layers in contracting G-fibers (Nishikubo et al., 2007; Mellerowicz et al., 2008; Baba et al., 2009). Here, we have identified *PtXTH5* as an XTH member specifically induced during G-layer biosynthesis, whereas majority of other XTH genes were downregulated (**Figure 7A**). Some of these downregulated XTHs (*PtXTH21*, *26*, *35*, *41*) showed increased expression in TW compared to NW during S-layer formation. These are the candidates for G- to S-layer binding function in TW (Nishikubo et al., 2007; Mellerowicz et al., 2008; Baba et al., 2009). The suppression of the majority of *XTH* genes at later stages of TW formation could be understood as restricting excessive transglucosylation activity that would lead to relaxation of layer binding and loss of tension. We also identified putative xyloglucan α-xylosidases (GH31), α-fucosidases (GH95), and a β-galactosidase *PtBGAL10* (GH31) (**Supplementary Table S11**), which were suppressed during S- and G-layer biosynthesis (**Figure 7A**). These results suggest that xyloglucan side chains are not excessively trimmed during S- and G-layer formation in TW and the main xyloglucan modification is transient transglucosylation.

Xylans, the most abundant hemicelluloses of the S-layers in dicotyledons, have either not been detected in G-layers (Bowling and Vaughn, 2008; Decou et al., 2009; Gorshkova et al., 2015) or detected with certain antibodies at a low level (Kim and Daniel, 2012; Higaki et al., 2017). A specific form of glucuronoxylan might exist in the inner G-layer (Guedes et al., 2017; Higaki et al., 2017). Glucuronoxylan is synthesized by synthase complexes specialized for P- and S-layer biosynthesis including IRX10 or IRX10L (GT47) and accessory proteins IRX9 or IRX9L and IRX14 (GT43) (Anders et al., 2023). Glucuronosyl substitutions are added by GUX proteins (GT8), whereas the reducing end tetrasaccharide sequence is made by GATL1/PARVUS (GT8), GAUT12/IRX8 (GT8), FRA9 (GT106), and FRA8 (GT47) (Ye and Zhong, 2022; Zhong et al., 2023). P-layer glucuronoxylan has pentose substitutions added to the backbone by xylosyl and/or arabinosyl transferases (GT61) (Zhong et al., 2025). Previous analyses of *Populus* TW provided inconsistent data regarding glucuronoxylan biosynthesis (Andersson-Gunnerås et al., 2006; Bygdell et al., 2017). The present analysis did not detect significant changes in the expression of *IRX10*, *IRX9*, and *IRX14* homologs or genes encoding reducing-end biosynthetic GTs during G-layer formation, although many of them showed declining trends (**Figure 7B**). The exceptions were *PtGALTL2-A*, which was suppressed, and *PtFRA9A*, which was induced. Among *GUX* genes, homologs of *GUX1* and *GUX3* (*PtGUX1-A* and *PtGUX3*) responsible for xylan glucuronidation with specific patterns in S- and P-layers, respectively (Mortimer et al., 2015), were suppressed, whereas a homolog of an uncharacterized *GUX4* gene (*PtGUX4-A*) was induced during G-layer formation (**Figure 7B**). The biggest expression changes were observed among xylosyl- and/or arabinosyltransferases decorating the xylan backbone (GT61): whereas two of them (*PtXXAT1* and *2*) were most highly expressed at the beginning of the developmental series in NW and TW, the others (*PtX2AT1*, *PtXXAT2-like*) were induced during G-layer formation similar to *PtGUX4-A*. This suggests that there might be a specialized machinery for G-layer glucuronoxylan biosynthesis, generating a specific glucuronoarabinoxylan structure found in the inner G-layer (Guedes et al., 2017).

We also observed regulated expression of carbohydrate esterase 6 (CE6) members recently identified in *Populus* (Kumar et al., 2019) and encoding xylan or mannan acetyl esterases (Mai-Gisondi et al., 2017): *Potri.001G300800* was suppressed and *Potri.014G022700* induced during G-layer formation (**Figure 7B**), indicating the presence of post-synthetic xylan (or mannan) deacetylation machinery specific for G-layer maturation.

Mannans, which are ubiquitous cell wall components, were detected in the G-layers in *Populus* TW (Nishikubo et al., 2007; Kim and Daniel, 2012; Guedes et al., 2017), but mannose content was reportedly approx. three times lower compared to NW (Gorshkova et al., 2015). Correspondingly, the expression of *PtCSLA1/PtGT2A*, encoding the main *Populus* mannan synthase (Guevara-Rozo et al., 2026), was reduced in TW compared to that in NW (Andersson-Gunnerås et al., 2006). Current analysis found decreased expression of not only *PtCSLA1* but also *PtCSLA3* and *4* and stable expression of *PtCSLA2* and *5* during G-layer biosynthesis (**Figure 7C**). Homologs of *MSR1*-like genes (GT106) participating in mannan biosynthesis (Voiniciuc, 2022) showed a stable expression during NW and TW development, whereas all three putative mannan α-galactosyl transferases identified in the *MUCI10* clade of family GT34 (Voiniciuc, 2022; Kumar et al., 2019) showed a trend for transient suppression at T3. These results support a reduced capacity for mannan biosynthesis during G- compared to S-layer formation.

Mannan backbone is post-synthetically hydrolyzed or transglycosylated either intracellularly or *in muro* by endo-β-mannanases/transglycosylases from GH5 family, subfamily 7 (GH5_7) (Jacobson et al., 2026). Here, we found *PtMAN7* (*AtMAN6*) suppressed and *PtMAN8* (*AtMAN2*) induced during G-layer formation, whereas *PtMAN4* and *6* (both similar to *AtMAN5*) showed a broad peak of expression covering the main part of G-layer biosynthesis (**Figure 7C**). These patterns implicate *PtMAN4*, *6*, and *8* in G-layer formation. These mannanases are atypical and membrane-bound with a proposed function in mannan biosynthesis or signaling via mannooligosaccharides (Zhao et al., 2013a; Jacobson et al., 2026).

#### 2.4.6 Unknown function of GT and GH candidates for the generation of novel structures of G-layer polymers

Analysis of CAZyme gene expression during G-layer synthesis identified several upregulated GT and GH genes (**Supplementary Figure S2**), which have not been characterized so far but likely have cell wall-related functions. They include three members of poorly characterized clades of the GT47 family (*PtGT47B-1/Potri.013G067400*, *GT47_18/Potri.007G116800*, and *GT47_28/Potri.006G239000*) (Kumar et al., 2019) and one member of the CSLG clade of the GT2 family (*PtCSLG4*/*Potri.003G142500*). *PtGT47B-1/Potri.013G067400* belongs to clade B of GT47 that has recently been shown to include the RG-I galactan-initiating enzymes GalTi (Corulli et al., 2026), and considering the importance of RG-I for the G-layer, it likely encodes GalTi. The CSLG clade is closely related to CSLM1 and CSLM2 clades found in several dicot species but not in *Arabidopsis*, which might function in AGP biosynthesis as β-glucuronosyltransferases (Hassan et al., 2025). *PtGT14_13* (*Potri.013G145500*) from an uncharacterized clade of the GT14 family is also likely an AG-II-related GT, along with two genes from an uncharacterized clade of the family GT77 (*GT77_11*/*Potri.010G099400* and *GT77_8*/*Potri.015G029200*). The two upregulated GH31 members (*GH31_6*/*Potri.001G442800* and *GH31_11*/*Potri.011G154200*) are classified as apoplastic α-glucosidases, but their substrate specificity has not so far been determined.

#### 2.4.7 Substrate supply for Golgi polysaccharide biosynthesis

The robust synthesis of RG-I and AG-II during G-layer formation necessitates a steady influx of cytosolic nucleotide sugars into the Golgi apparatus. This transport is mediated by nucleotide-sugar transporters (NSTs), representing a critical regulatory checkpoint for polysaccharide assembly. Eighteen NSTs were expressed in NW and TW (Table S15). Aligning with the specific polysaccharide demands of the G-layer, the transcriptomic data revealed a coordinated shift in NST expression. Homologs of UDP-rhamnose/UDP-galactose transporters (*URGTs*), which are essential for supplying substrates for RG-I and galactan biosynthesis (Rautengarten et al., 2014), were significantly induced during G-layer formation. Specifically, expression of one *URGT1* homolog (*Potri.005G260300*) strongly increased from the onset of G-layer formation, maintaining high expression through the main deposition phase (T3–T5) before ultimately peaking at the final stage (T7), while a *URGT3* homolog (*Potri.005G086700*) showed a broad peak during the main deposition phase (**Supplementary Table S15**). Furthermore, two homologs of *GONST5* (*Potri.002G084500* and *Potri.005G177200*) display prominent upregulation peaking at T4 and T5, distinguishing them from other stable or lowly expressed *GONST* members. Although specificity of *GONST5* is currently unknown, the characterized members of the *GONST* family are Golgi-localized GDP-mannose transporters involved in N-glycosylation of glycosylphosphatidylinositol (GPI)-anchored proteins (Baldwin et al., 2001; Jing et al., 2021). The induction of these *GONST5* homologs is therefore likely required to support the massive post-translational modification and membrane anchoring of G-layer-specific proteins, such as the highly upregulated fasciclin-like arabinogalactan proteins (FLAs).

Conversely, the import of UDP-xylose—required for xylan and xyloglucan biosynthesis—is mediated by UDP-xylose transporters (UXTs) (Ebert et al., 2015). In agreement with the broader suppression of the xylan biosynthetic machinery during G-layer differentiation, the highly expressed *UXT1* (*Potri.004G211900* and *Potri.009G011100*) and *UXT2* (*Potri.019G128900*) homologs demonstrate their highest expression during NW biosynthesis and S-layer formation (T1–T2) in TW, followed by a decline during G-layer deposition (T4–T7) (**Supplementary Table S15**). These contrasting profiles confirm that the massive transcriptional reorganization during tension wood formation actively fine-tunes the Golgi substrate supply chain to directly support G-layer-specific cell wall construction.

### 2.5 Expression of genes involved in lignin biosynthesis

Arguably, the most defining feature of the G-layer that distinguishes it from the S- and compound middle lamella (CML) layers is the severe reduction or absence of lignin (Fagerstedt et al., 2014). Compared to NW, TW lignin has a higher proportion of syringyl (S) and *p*-hydroxyphenyl (H) units and a lower proportion of guaiacyl (G) units (Joseleau et al., 2004). The change in S/G ratio is attributed to the increased fibers/vessel elements ratio. In some species, lignin was detected in the outer or inner part of the G-layer (Higaki et al., 2017). Lignification of the CML and S cell wall layers continues throughout S- and G-layer deposition (Yoshinaga et al., 2012), but it is unclear if this requires a continued supply of monolignols or only involves polymerization of monolignols already present in cell walls. Past expression analyses in *Populus* TW demonstrated that transcripts involved in monolignol biosynthesis were unanimously downregulated compared to NW or opposite wood (Andersson-Gunnerås et al., 2006; Chen et al., 2015), yet many corresponding proteins increased in abundance during late tension wood formation (Bygdell et al., 2017).

Based on phylogenetic analyses and previous studies (Ehlting et al., 2005; Hamberger et al., 2007; Sundell et al., 2017), we identified 116 lignin biosynthesis-related genes expressed during NW and TW formation (**Supplementary Table S16**), representing 13 different families of enzymes from the monolignol biosynthetic pathway, laccases, and peroxidases. Majority of the main (most highly expressed) family members showed the highest expression during S-layer formation in NW and TW, sometimes extending to the beginning of the G-layer formation (T3) in TW and then typically declining, but the decrease in transcripts during G-layer formation was subtle, and statistically significant changes usually were not observed (**Figure 8**). Such patterns are typical for the S-layer biosynthetic genes (**Figure 2B**). Few genes had stable expression after reaching a maximum in T2/T3 (e.g., *PtPAL1*, *PtPAL2 PtPAL3*, *PtCAD1*, *PtPRX1*), and others showed stable expression in NW and TW series (e.g., all highly expressed CCR and CAD genes). A few other genes showed a tendency for increased expression (*PtHCT11*) or *de novo* induction during the late stage of S- and/or G-layer formation (*CAD Potri.001G372400*, *PtPRX9*, *PtPRX30*, *PtPRX86*). This could be related to the production of monolignols and lignification or to the formation of related compounds known to be synthesized by the phenylpropanoid pathway enzymes, such as condensed tannins and flavonoids (Kao et al., 2002; Harding et al., 2002; Tsai et al., 2006; Vanholme et al., 2012; Li et al., 2015). These compounds are expectedly synthesized during late stages of xylem cell differentiation as part of wood extractives. Overall, the current transcriptomic analysis did not reveal any strong decrease in transcripts of monolignol biosynthetic genes during G-layer formation, suggesting that these transcripts are not specifically degraded but rather persist during the entire process of cell wall formation.

**Figure 8:**
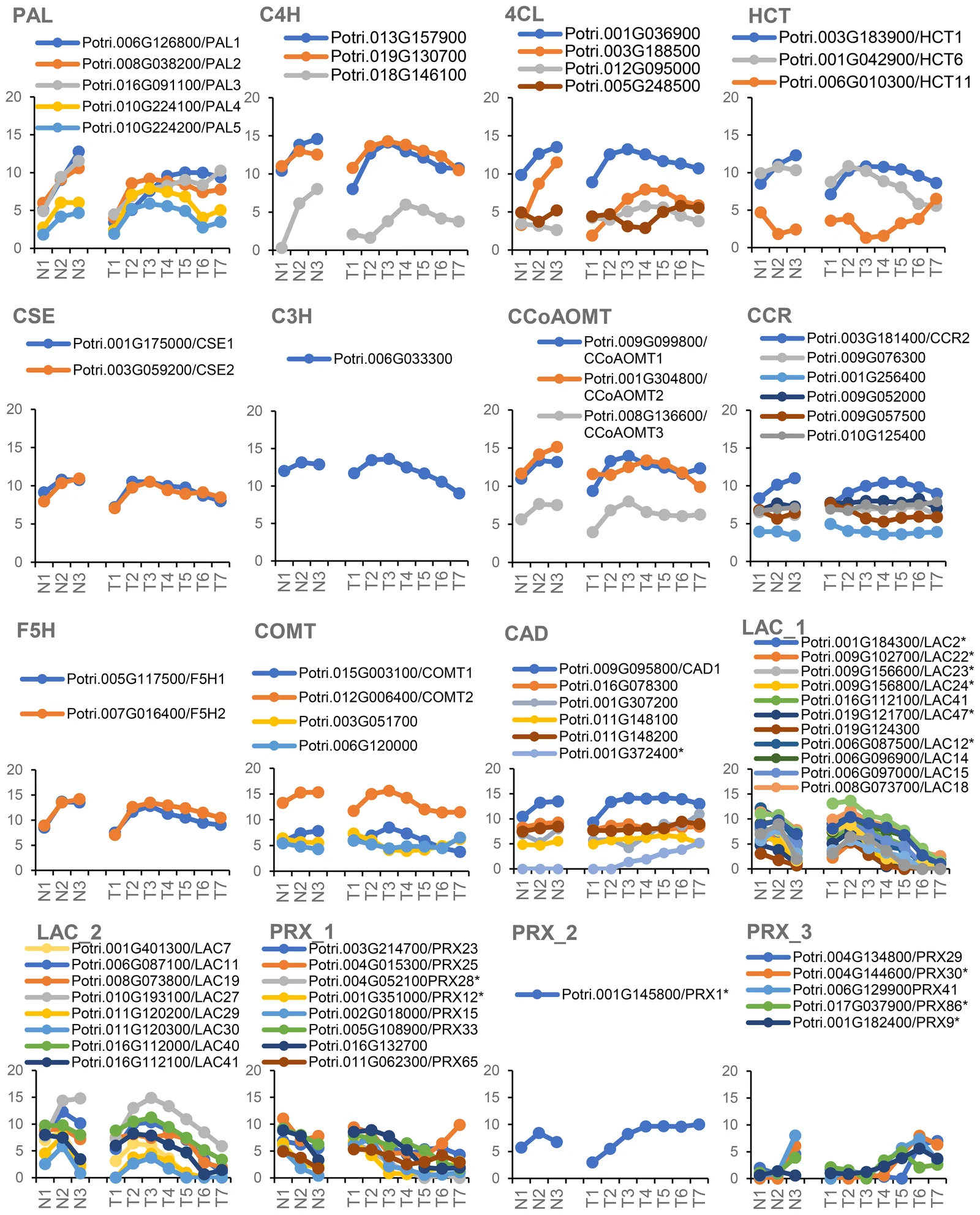
Expression of genes involved in lignin biosynthesis during normal wood (N1–N3) and tension wood (T1–T7) formation. Gene expression profiles represent an average VST from two biological replicates. Genes with stable and low expression (VST < 5) were not included. All lignin biosynthetic genes are shown in **Supplementary Table S16**. Abbreviations: PAL, phenylalanine ammonia-lyase; C4H, cinnamate 4-hydroxylase; 4CL, 4-coumarate:CoA ligase; HCT, hydroxycinnamoyl-CoA shikimate/quinate hydroxycinnamoyl transferase; CSE, caffeoyl shikimate esterase; C3H, coumarate 3-hydroxylase; CCoAOMT, caffeoyl coenzyme A O-methyltransferase; CCR, cinnamoyl-CoA reductase; F5H, ferulate 5-hydroxylase; COMT, caffeic acid O-methyltransferase; CAD, cinnamyl alcohol dehydrogenase; LAC, laccase; PRX, peroxidase. Stars indicate differentially regulated genes during the early and/or late phase of G-layer synthesis.

In contrast, many laccase-encoding genes (the expression group LAC_1 in **Figure 8**), including homologs of *LAC4* implicated in xylem lignification in *Arabidopsis* (Zhao et al., 2013b; Yi Chou et al., 2018), *PtLAC2* and *PtLAC4*, were significantly downregulated. Also, *PtLAC11* (*Potri.006G087100*), homologous to *AtLAC17*, also known as *PtLAC110*, regulating lignin polymerization in *Populus* (Ranocha et al., 2002; Bryan et al., 2016), showed a clear trend of downregulation in N3 and T6–T7 samples (the expression group LAC_2 in **Figure 8**). Peroxidases have been also shown to control lignification (Sasaki et al., 2004) and are considered to have a more important role than laccases in G-lignin polymerization (Barros et al., 2015). In *Arabidopsis*, *PRX25*, *56*, *64*, and *72* were shown to control cell wall lignification. In *Populus*, *PtPrxA3a3* (*Potri.003G2148*, not expressed in our samples) was demonstrated to function in cell wall assembly (Li et al., 2003). Based on expression in NW, *PtPRX39*, a homolog of *AtPRX25*, could be the main peroxidase involved in xylem lignification as its expression peaked during S-layer formation (Sundell et al., 2017). *PtPRX39* was lowly expressed in TW and exhibited a typical S-layer expression pattern (**Supplementary Table S16**), similar to several other peroxidases, which were significantly suppressed during late G-layer formation (expression pattern PRX_1 in **Figure 8**), supporting their role in TW lignification. Considering the relatively low frequency of vessel elements in TW, the role of peroxidases in TW lignification is expectedly lower compared to that of laccases. Other expression patterns seen among the peroxidases (described above) suggest a different function than cell wall lignification. Overall, these observations suggest that a tighter control at the transcript level is exerted for the lignin polymerization-controlling laccases and peroxidases than for the monolignol biosynthetic genes.

### 2.6 Identification of co-expression modules associated with G-layer biosynthesis

To identify transcriptional networks during TW formation, hierarchical clustering was performed for 22,363 expressed genes, and the resulting dendrogram was split into 90 co-expression clusters (**Supplementary Figure S3**; **Supplementary Table S2**). Functional enrichment [MapMan, Gene Ontology (GO), and PFAM; **Supplementary Table S17**–**S19**] was then used to characterize the biological processes associated with each cluster. These clusters were subsequently grouped into four categories: (i) clusters with stable expression across NW and TW development, (ii) clusters with the highest expression during primary–secondary wall transition, (iii) clusters induced during S-layer biosynthesis, and (iv) clusters induced during G-layer biosynthesis (**Figure 9**; **Supplementary Table S20**).

**Figure 9:**
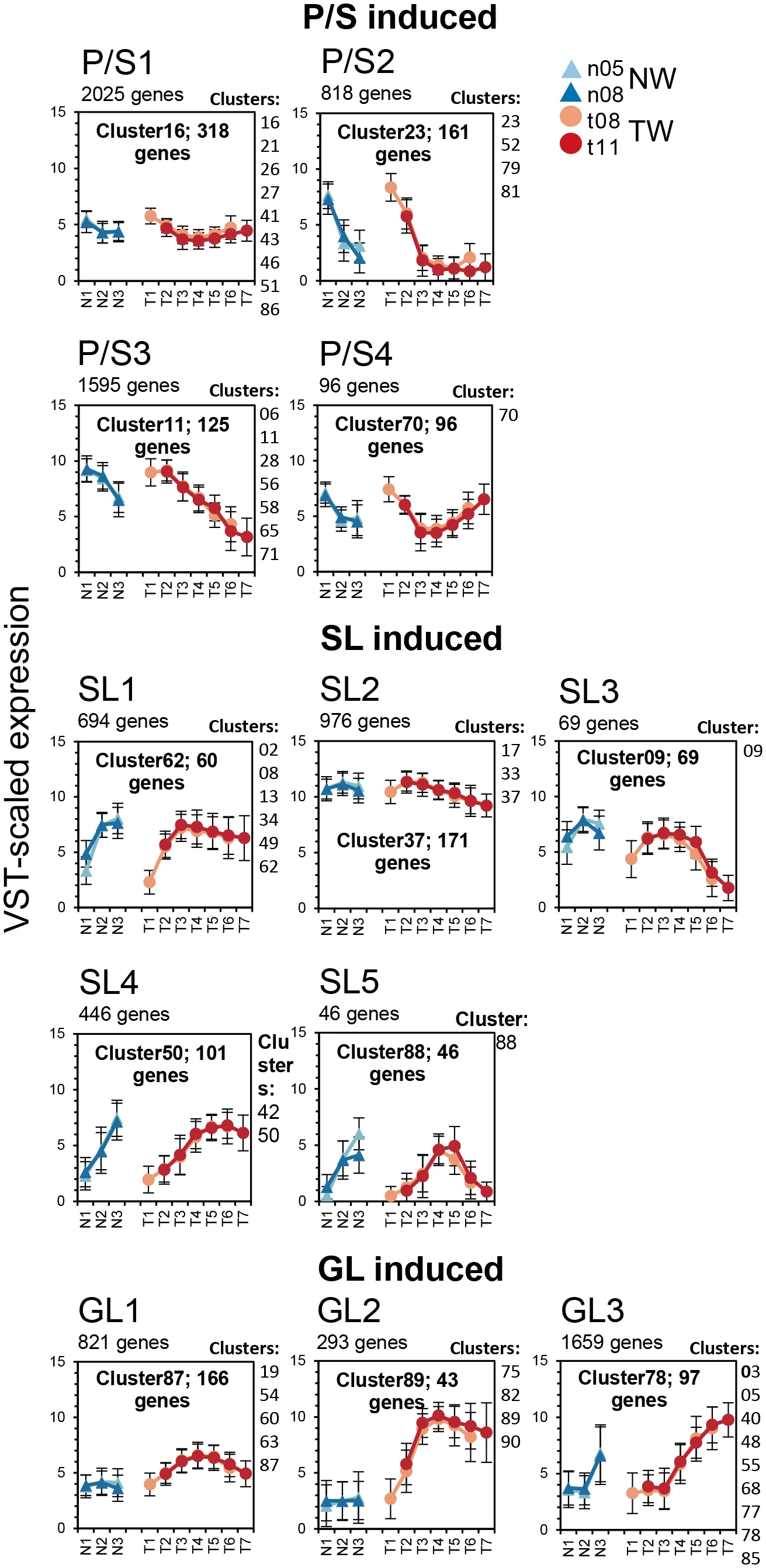
Selected types of gene expression patterns in normal wood (NW) and tension wood (TW) samples displaying clear trends during primary–secondary wall transition (P/S), main S-layer (SL), or G-layer (GL) formation. The co-expression analysis identified 90 clusters (shown in **Supplementary Figure S3**), which were grouped into developmentally relevant types of patterns representing genes induced during P/S transition and SL or GL biosynthesis. The plots display VST-scaled gene expression across the developmental series (N1–N3 and T1–T7) for two NW-forming (n05, n08) and two TW-forming (t08, t11) trees. Mean ± SE is shown for each representative cluster, calculated from all genes assigned to that cluster. The side panel provides an overview of other clusters exhibiting comparable expression profiles. Gene lists for each cluster are shown in **Supplementary Table S2**.

#### 2.6.1 Category I: clusters with stable expression

Category I clusters (S1–S5; **Supplementary Table S20**) comprise 13,096 genes (59% of all expressed genes) whose expression remained essentially stable across the developmental series in both NW and TW. These clusters are enriched in genes associated with housekeeping and general cellular functions, such as general metabolism, DNA and chromatin maintenance, mitochondrial functions, transport, and protein and RNA biosynthesis. These activities are required throughout wood formation and are not specifically linked to S- or G-layer biosynthesis.

#### 2.6.2 Category II: clusters induced during primary to secondary wall transition

Category II clusters (4,534 genes, 20% of all expressed genes) include genes with the highest expression at the beginning of the developmental series in NW and TW samples and either very lowly expressed later (**Figure 9**; P/S2, P/S3) or induced at the late developmental stages in TW (**Figure 9**; P/S1, P/S4, P/S5). Clusters P/S2-P/S3 are enriched in functions related to cell division, cell expansion, primary wall biosynthesis and modification, phloem functions (raffinose and callose metabolism), photosynthesis, innate immune response, and stress. Clusters P/S1, P/S4, and P/S5 include TFs mediating auxin responses (IAA/AUX), calcium signaling and stress responses (including apoptosis- and salicylic acid-related genes) together with regulatory genes (RLKs and ubiquitination machinery), amino acid and oligopeptide transporters, and proton pumps.

#### 2.6.3 Category III: clusters induced with secondary wall biosynthesis

Category III clusters (1,960 genes, 8.8% of all expressed genes) are represented by patterns SL1–SL5 (**Figure 9**), containing genes whose expression is induced during S-layer biosynthesis in both NW and TW and maintained throughout the developmental gradient or suppressed at the late TW stages. These clusters are enriched in genes involved in lignin and S-layer cellulose biosynthesis (including secondary wall *CesAs*); hemicellulose biosynthesis; callose metabolism; cell wall protein biosynthesis; *GT47*, *GH9*, and *GH5* families; laccase, peroxidase, amino acid, and lipid metabolism; cytoskeleton organization; a wide range of transport processes; stress- and hormone-related pathways; and DNA replication/repair/epigenetics, as well as multiple TF families (*LOB-domain*, *MYB*, *WRKY*, *bZIP*, *NAM*, *HLH*).

Overall, the expression profiles of category II and III genes show that a substantial fraction of the secondary wall program remains unchanged between NW and TW, highlighting the need for high spatial resolution transcriptome analyses across the TW developmental gradient to resolve TW-specific induction and maintenance networks.

#### 2.6.4 Category IV: G-layer-associated clusters

Category IV (2,773 genes, 12% of all expressed genes) comprises clusters with G-layer-associated, TW-specific expression (**Figure 9**, patterns GL1–GL3). These clusters were enriched in G-layer-related cell wall biosynthetic and remodeling genes (enzymes of cell wall precursor biosynthesis, galactosyltransferases *GT31/GT92*, including *PtGALS2-A*; rhamnogalacturonan lyases, including *PtPL4_4*; *FLA12s*), stress-related genes, transporters for amino acids, and regulatory components, including MAP kinases, oligopeptides, ethylene biosynthesis genes, ABA metabolism, and genes encoding TFs with GRAS (SCL18-like), WRKY, NAM, helix–loop–helix, and BRLX domains. The BRLX-domain gene corresponds to *Potri.018G064900*, homologous to *BRLX4*, which encodes a protein-mediating gravity response in *Arabidopsis* stems through interaction with LAZY1 (Che et al., 2023), suggesting a shared gravity-sensing pathway between TW and primary tissues. Consistent with this, similar changes in LAZY1-associated protein phosphorylation were detected in developing TW and gravitropically stimulated roots (Hsieh et al., 2025).

Some genes in clusters with GL1–GL3 expression patterns were induced *de novo* during G-layer formation (**Supplementary Table S21**). We used this list to identify G-layer-specific genes by excluding genes expressed in tissues where G-layers are unlikely to occur, such as seeds or roots. This resulted in eight genes considered as G-layer specific (**Table 1**). Among them were two cell wall-related genes, FLA12-like and PtXTH5, which had already been discussed. We also identified a LIM family gene *δLIM2a* from a clade that is absent in *Arabidopsis* and monocots and has been implicated in TW biosynthesis (Arnaud et al., 2007). The remaining genes are known for their various regulatory functions, which could have been recruited to control G-layer formation. This includes two TFs, a homolog of *SCL18* regulating branching (Yuan et al., 2024) and *AGL29* involved in ovule development (Bemer et al., 2010), and the signaling-related gene *RIC7*, which negatively regulates light-mediated stomatal opening by affecting vesicle trafficking (Hong et al., 2016).

**Table 1:** G-layer-specific genes.

*Populus trichocarpa* v 3 gene ID	N1-N3	T1	T2	T3	T4	T5	T6	T7	Immanen et al. (2017)			Sundell et al. (2015)				AGI	Ath symbol/description	Poplar name
									Phloem	Cambium	Xylem	Flower	Leaves	Roots	Seeds			
Potri.019G121300	*n*	*n*	3	6	7	6	6	5	*n*	*n*	*n*	*n*	*n*	*n*	*n*	AT5G60490	FLA12/FASCICLIN-like AGP	
Potri.006G160700	*n*	*n*	3	3	4	2	2	2	2	*n*	1	0	2	*n*	*n*	AT4G28850	XTH26/xyloglucan endotransglucosylase	XTH5
Potri.015G008300	*n*	*n*	2	3	4	5	4	4	2	*n*	1	1	*n*	*n*	*n*	AT1G55580	SCL18/GRAS family TF	
Potri.004G131100	*n*	*n*	*n*	1	2	5	5	6	1	*n*	*n*	*n*	*n*	*n*	*n*	AT2G34440	AGL29/AGAMOUS-like TF	
Potri.008G185800	*n*	*n*	1	4	6	6	6	5	1	*n*	1	1	1	*n*	*n*	AT1G67856	RING/U-box superfamily	
Potri.001G107100	*n*	*n*	1	3	4	4	3	2	1	*n*	*n*	*n*	*n*	*n*	*n*		/LIM protein	δLIM2a
Potri.002G035500	*n*	*n*	1	4	6	7	6	6	1	*n*	*n*	1	*n*	*n*	*n*	AT4G28556	RIC7/PAK-box/P21-Rho-binding protein	
Potri.006G215900	*n*	*n*	*n*	1	4	5	6	5	1	*n*	0	*n*	*n*	*n*	*n*	AT4G02750	SSTPR/Tetratricopeptide repeat protein	

Data show normalized expression. AGI identification was manually curated based on phylogenetic analyses.

*n*, not expressed.

Taken together, this categorization reveals four major expression regimes: stable, primary to secondary wall transition-associated, secondary wall-associated, and G-layer-associated, highlighting TW-specific co-expression modules and candidate regulators that likely define the core gene network controlling G-layer biosynthesis.

### 2.7 Identification of candidate genes for TW regulatory networks

#### 2.7.1 Hormonal signaling

Phytohormones like ethylene, gibberellins, auxins, and brassinosteroids have been implicated as central regulators of TW development and its transcriptional reprogramming (Andersson-Gunneras et al., 2003; Gerttula et al., 2015; Felten et al., 2018; Seyfferth et al., 2019; Wessels et al., 2019; Du et al., 2020; Jin et al., 2020). To capture the main hormonal putative regulators involved in early responses leading to the synthesis of the G-layer, we focused our analysis on hormone-related genes with a TW-specific expression pattern represented by GL1 and GL2 (**Figure 9**). These clusters were enriched in genes related to ethylene and 2-oxoglutarate-dependent dioxygenase activity related to ethylene, jasmonate, and gibberellin biosynthesis.

We detected early induction of ethylene biosynthesis genes including ACC oxidases similar to *AtCO4* (*Potri.014G159000*) and *AtCO5 (Potri.005G182700*, *Potri.002G078600*) and signaling genes, e.g., homologs of *AtEBF1* (*Potri.006G068500*), *AtCTR1* (Potri.010G234700), *AtEIN3* (*Potri.008G011300*, *Potri.010G247500*), and *AtERS1* (*Potri.002G201500*) (**Figure 10A**; **Supplementary Table S22**), consistent with genetic and transcriptomic studies showing that ethylene perception and signaling are required and sufficient for the development of fully functional tension wood and G-layer biosynthesis even in the absence of the gravitropic stimulus (Love et al., 2009; Vahala et al., 2013; Felten et al., 2018; Seyfferth et al., 2019; Wessels et al., 2019). Previous time-course expression analysis (Yu et al., 2021) found downregulation of these *AtEIN3* homologs after stem bending (**Supplementary Table S23**), highlighting differences between bulk tissue analysis and analysis of defined developmental stages.

**Figure 10:**
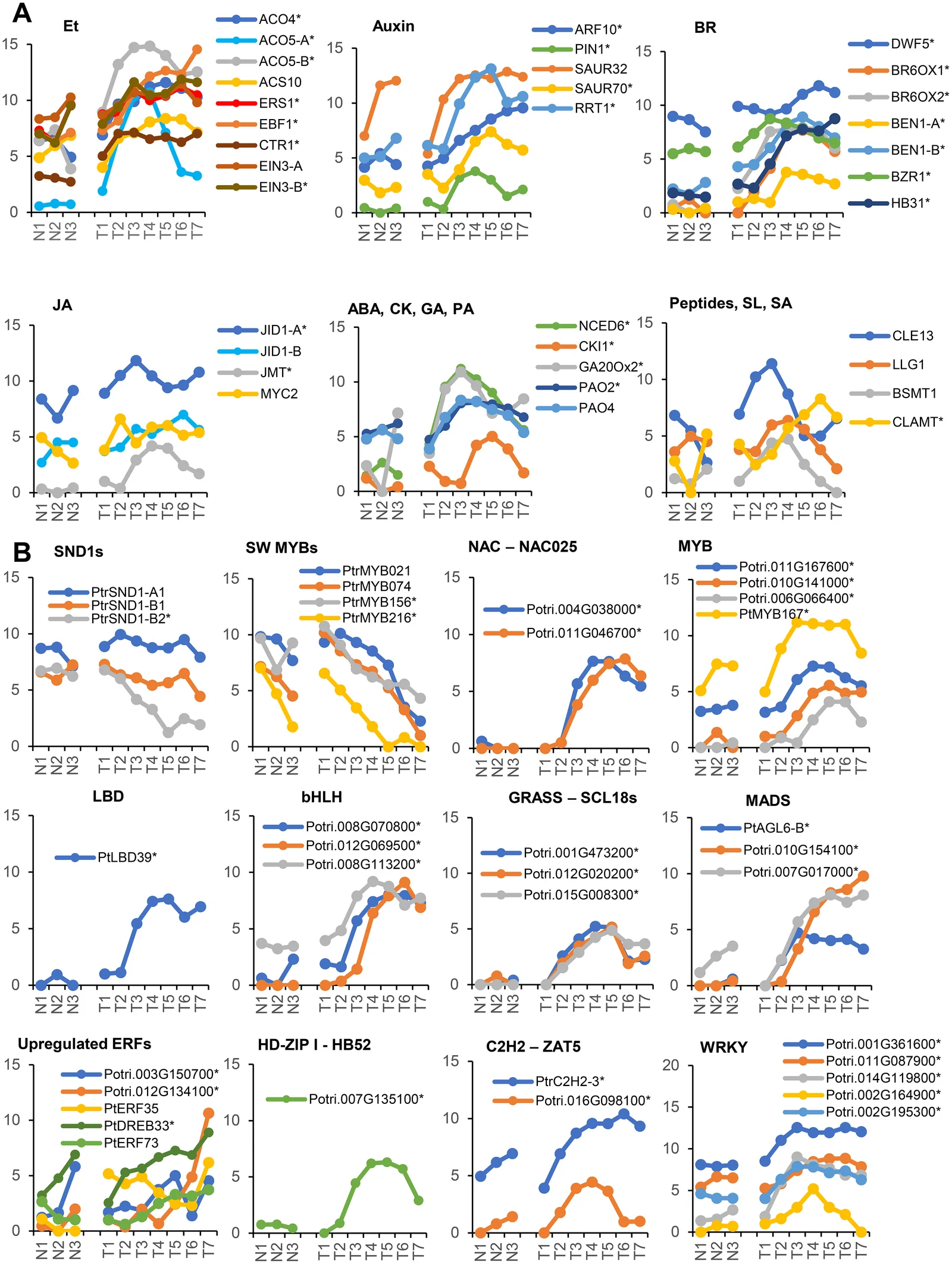
Expression of hormone-related genes **(A)** and transcription factors **(B)** induced during G-layer biosynthesis. Average VST expression for two biological replicates (n05/n08 and t08/t11) is shown. Stars indicate differentially regulated genes during the early and/or late phase of G-layer synthesis. Potri codes are shown in **Supplementary Tables S22**, **S23**. Et, ethylene; BR, brassinosteroids; JA, jasmonates; ABA, abscisic acid; CK, cytokinins; GA, gibberellins; PA, polyamines; SL, strigolactones; SA, salicylic acid.

Jasmonates are known as positive regulators of cambial activity (Sehr et al., 2010), which is one of the defining features of TW. Here, we observed upregulation of the master regulator in jasmonic acid signaling—a homolog of *AtMYC2* (*Potri.002G176900*)—and jasmonate biosynthetic genes homologous to *AtJID1* (*Potri.017G135800*, *Potri.010G073300*) and *AtJMT* (*Potri.007G021300*) during the early stage of G-layer formation (**Figure 10A**; **Supplementary Table S22**). This aligns with recent work in hybrid aspen flexure wood reporting elevated jasmonate levels and signaling in mechanically stimulated stems, which formed TW (Urbancsok et al., 2023). Although *MYC2* homologs have not yet been directly demonstrated to control biosynthesis of the G-layer, studies in *Arabidopsis* indicate that MYC2/MYC4 regulate fiber secondary cell wall thickening and cell wall chemistry, integrating light and stress signals with jasmonate signaling (Im and Son, 2025). The *MYC2* homolog *Potri.*002G176900 was also reported as being upregulated from day 3 in the time-course transcriptomic analysis of TW induction (Yu et al., 2021). These reports, together with our transcriptomics data, support a potential role of jasmonate signaling in both aspects of TW formation, cambial activation, and cell wall remodeling.

The induction of brassinosteroid biosynthesis-related genes such as homologs of *AtDWF5* encoding a sterol delta-7-reductase (*Potri.010G253200*), brassinosteriod 6-oxidases homologous to *AtBR6OX1* (*Potri.004G117700*) and At*BR6OX2 (Potri.017G099000*), and homologs of *AtBEN1* (*Potri.002G147400*, *Potri.002G147700*), as well as key TFs mediating brassinosteroid responses *AtBZR1* (*Potri.007G030700*) and *AtHD31* (Potri.013G108900) at the onset of G-layer biosynthesis (**Figure 10A**; **Supplementary Table S22**), is in line with functional studies demonstrating that enhanced brassinosteroid levels promote secondary growth and G-layer thickening in poplar (Jin et al., 2020; Du et al., 2020) and are involved in flexure wood formation (Urbancsok et al., 2023).

Considerable evidence implicates gibberellins in TW biosynthesis (Funada et al., 2008; Gerttula et al., 2015; Wang et al., 2017). Our analysis identified a homolog of *AtGA20Ox2* (*Potri.015G134600*), encoding a critically important oxidase for generating active gibberellins, highly upregulated at the onset of G-layer biosynthesis (**Figure 10A**; **Supplementary Table S22**), consistent with the involvement of gibberellins in G-layer differentiation. Whether cytokinins also participate in this process is not known. Only one gene related to cytokinin perception was found among early induced genes (*Potri.013G009700* similar to *AtCKI1*). *AtCKI* is implicated in female gametophyte development and vegetative growth (Deng et al., 2010); its involvement in TW differentiation warrants further study.

Regulation of auxin transport by the PIN3 auxin efflux carrier has been considered as a regulatory step mediating gravity sensing and TW formation (Gerttula et al., 2015). Here, we found that the homolog of *AtPIN1* (*Potri.006G037000*), along with the homologs of auxin-regulated genes *AtARF10* (*Potri.004G211700*), *AtSAUR32* (*Potri.012G023400*), and *AtSAUR70* (*Potri.018G063400*), were specifically induced at the onset of G-layer formation (**Figure 10A**; **Supplementary Table S22**). These data support the idea of altered auxin transport during TW development. *AtSAUR32* is implicated in ABA-regulated responses (He et al., 2021). In our dataset, upregulation of its homolog coincided with the upregulation of 9-cis-epoxycarotenoid dioxygenase similar to *AtNCED6* (*Potri.003G176300*), suggesting a rise in ABA at the onset of G-layer formation. These data support the positive involvement of ABA in G-layer formation as suggested by an earlier study in *Catalpa bungei* (Xiao et al., 2020).

We also identified homologs of genes related to polyamine, salicylic acid, and strigolactone metabolism, including polyamine oxidases similar to *AtPO2* (*Potri.005G207300*) and *AtPO4* (*Potri.T060000*), and the homolog of benzoic acid/salicylic acid methyltransferase *AtBSMT1* (*Potri.019G022100*) induced at the onset of G-layer formation as well as a carlactonic acid methyltransferase *CLAMT* (*Potri.007G021400*) induced at a later stage (**Figure 10A**, **Supplementary Table S22**). Moreover, we found evidence for peptide hormone participation in G-layer induction, identifying homologs of *AtCLE13* (*Potri.008G115600*) and *AtLLG1* (*Potri.013G132000*) among the highly and early upregulated genes. These findings implicate these hormones in TW response for the first time.

Collectively, these observations reinforce the view that TW formation emerges from a coordinated crosstalk between ethylene, jasmonate, ABA, gibberellin, and brassinosteroid signaling networks in wood-forming tissues and suggest new candidate regulators, including polyamines, peptide hormones, salicylic acid, and strigolactones in the induction of the G-layer.

#### 2.7.2 Transcription factors

The vast changes in gene expression that occur during TW formation (**Supplementary Figure S3**, **Supplementary Table S2**) are likely coordinated by a network of upstream TFs. Identifying TFs involved in switching on G-layer formation has long been a goal in this field, partially due to its potential for engineering wood with desirable properties for biofuel production. Out of 2,465 TFs from PlantTFDB (https://planttfdb.gao-lab.org/index.php?sp=Ptr), 1,288 were present in our list of expressed genes (**Supplementary Table S23**). It is well established that the induction of the S-layer is regulated by two or more tiers of master TFs forming a regulatory network; the first tier SND1 induces the second tier MYB TFs such as *Pt*MYB021 and 074, which then activate S-layer biosynthetic genes (Zhong et al., 2008, 2011; Chen et al., 2019). If this network was also involved in G-layer induction, one would expect its strong upregulation during the early stages of G-layer formation. All three *SND1* genes and their main target *MYBs* in our dataset (*PtMYB021*, *074*, *156*, *216*) showed the highest expression at the primary/secondary wall transition and a declining trend during G-layer biosynthesis (**Figure 10B**), consistent with their function as S-layer master switchers not participating in the G-layer differentiation. This supports the concept of the G-layer as the tertiary rather than secondary cell wall. We then focused on TFs with expression patterns GL1 and GL2, where genes were sharply induced during early G-layer formation (T3–T4) and exhibited low expression throughout NW development (**Figure 9**). Overall, 68 upregulated transcription factors were identified in these clusters (**Supplementary Table S23**), and 30 of them exhibited striking upregulation at the onset of G-layer formation typical for the *de novo* induction pattern. Selected examples of these TFs are shown in **Figure 10B**. Two homologs of *AtNAC025*, *Potri.004G03800/PNAC048* and *Potri.011G046700*/*PNAC047*, and three MYB domain TFs were among the *de novo*-induced genes, including the homologs of *AtMYB82* (*PtMYB135/Potri.006G066400*), *AtMYB80* (*Potri.011G167600*), *AtMYB111* (*PtMYB035/Potri.010G141000*), and *AtMYB52* (*PtMYB167*/*Potri.012G039400*), which were strongly induced in T3 or later (**Figure 10B**). *PNAC047*, *PNAC048*, *PtMYB035*, and *PtMYB167* (but not *PtMYB135*) were reported to be upregulated in a time-course study of TW induction (Yu et al., 2021). These TFs are candidates for the G-layer transcriptional regulatory network by analogy to NAC and MYB TFs regulating secondary wall formation (Zhong et al., 2008, 2011).

*De novo*-induced TFs included also a LOB-domain transcription factor *PtLBD39* (*Potri.005G097800*), which is known as a master regulator of TW formation, highly induced after 5 days of stem bending (Yu et al., 2021), confirming the usefulness of our approach. The *Arabidopsis* homolog of *PtLBD39*, *LBD4*, is part of the *PXY* transcriptional network regulating procambial and cambial cell divisions (Smit et al., 2020). Three poplar bHLH transcription factors similar to *AtBHLH161* (*Potri.012G069500*) known to be involved in brassinosteroid signaling (Wang et al., 2009), *AtBHLH49* (*Potri.008G113200*), and *AtBHLH* (*Potri.008G070800*) also showed similar induction (**Figure 10B**). Interestingly, *AtBHLH49* is also regulated by *PXY* signaling and participates in the regulation of axillary meristem identity, governed by *SCL18*, NAC TFs CUP-SHAPED COTYLEDONS (CUC) and REVOLUTA (Tian et al., 2014). Three *SCL18* homologs, *Potri.001G473200*, *Potri.012G020200*, and *Potri.015G008300*, were strongly upregulated at the onset of G-layer formation (**Figure 10B**). Taken together, these observations suggest that a genetic network similar to the one controlling axillary meristem identity might operate during TW formation.

Unexpectedly, three homologs of *Arabidopsis* floral homeotic MADS-box genes, *APETALA1* (*AP1-2*/*Potri.010G154100*), *AP3* (*Potri.007G017000*), and *AGAMOUS-LIKE6* (*PtAGL6-b/PTAGL6-1/Potri.012G132600*), were strongly upregulated at the onset of G-layer formation (**Figure 10B**; **Supplementary Table S23**), and the *AGL29* homolog (*Potri.*004G131100) was found as G-layer specific (**Table 1**). These genes were found upregulated in TW in the time-course analysis (Yu et al., 2021). In *Arabidopsis*, these MADS-box genes form transcriptional complexes that induce different flower organs. However, the phylogenetic analysis revealed that *PtAGL6-b* belongs to a paralogous clade of *AtAGL6* named *AGL6-like*, represented only by some eudicot species (Viaene et al., 2010). The *AGL6-like* genes in these species are primarily expressed in vegetative organs, including tendrils that produce G-fibers (Meloche et al., 2007), in contrast to the *AGL6* genes expressed in flowers, supporting participation of *PtAGL6-b*, possibly in complex with other MADS TFs, in the regulation of G-fiber differentiation in TW.

Consistent with the key role of ethylene in TW formation, previous studies identified 17 ethylene response factors (ERFs) expressed in TW, which might be responsible for triggering the downstream ethylene response (Vahala et al., 2013). Four of them, namely, *PtERF30*, *34*, *35*, and *139*, were among the 47 ERFs expressed during NW and TW formation in our study (**Supplementary Table S23**). At least five ERFs (*PtERF35/Potri.008G210900*, *PtERF73/Potri.007G076800*, *PtDREB33/Potri.014G025200*, *Potri.003G150700*, and *Potri.012G134100*) were induced at various stages of TW formation (**Figure 10B**). These ERFs could participate in the regulation of different aspects of TW formation; for example, *PtERF35* could mediate increased cambial activity during TW formation (Love et al., 2009), as its overexpression increased stem diameter (Vahala et al., 2013). Two other *ERFs* that have not been previously associated with TW formation, *Potri.012G134100* and *PtERF73*/*Potri.007G076800*, were found induced during early G-layer formation in our analysis and are candidates for mediating ethylene signaling inducing the G-layer. Among potential downstream regulators of ethylene signaling, we identified a homolog of *AtHB52* (*Potri.007G135100*). *AtHB52* encodes a homeodomain-Zip class I TF that regulates the expression of auxin transporter gene downstream ethylene signaling, regulating growth and gravitropism (Miao et al., 2018). The poplar homolog could have the same function, as it was induced *de novo* at the onset of G-layer deposition (**Figure 10B**), matching the expression profile of *PIN1* (**Figure 10A**).

Several stress-related TFs were found strongly upregulated during G-layer formation, including WRKY TFs, homologs to *AtWRKY 20*, *35*, *43*, and *69* (**Figure 10B**). One of them (*Potri.001G361600*) was also identified as early induced in differentiating G-fibers in single-cell RNA sequencing analysis (Hsieh et al., 2025). Furthermore, two C2H2 zinc finger TFs, homologous to *AtZAT5*, which was recently implicated in the regulation of xylogenesis under salt stress (Feng et al., 2025), were induced early during G-layer formation.

Overall, our analysis confirmed a key TF involved in TW differentiation, *PtLBD39*, and revealed many novel TFs with similar expression patterns: strongly induced expression at the onset of G-layer formation and low expression at the earlier stage in TW and in NW (**Figure 10**; **Supplementary Table S23**). These TFs are likely part of the genetic network involved in G-fiber fate specification and differentiation during TW development. Only seven TFs among the TFs expressed in developing TW and NW (**Supplementary Table S23**) were listed as candidates for G-fiber fate regulation based on single-cell RNA sequencing analysis (Hsieh et al., 2025), highlighting the complementary nature of our high-resolution spatial analysis.

## 3 Concluding remarks

The high-resolution spatial transcriptome generated in this study fundamentally advances our understanding of tension wood formation by illuminating the major developmental and molecular programs leading to the formation of functional tension wood with high cellulose content and contractile properties in *P. tremula*. By combining tangential cryo-sectioning with precise developmental alignment based on cell wall thickening, we were able to reconstruct a continuous differentiation trajectory from early S-layer deposition to late G-layer maturation. This approach revealed that TW formation is not a gradual modification of the NW differentiation program but rather a similar process as NW formation during primary and secondary wall deposition, followed by a discrete developmental switch marked by a sharp transcriptomic reprogramming at the onset of G-layer deposition.

We found that G-layer formation constitutes one of the most extensive transcriptional reprograming known in wood development with almost 2,900 genes specifically regulated during early and late G-layer formation in TW. Genes expressed during NW and TW formation fall into large co-expression clusters enriched for functions including carbohydrate metabolism, protein trafficking, oxidative stress responses, and hormone-related signaling. Importantly, while this analysis confirmed major molecular players involved in TW development revealed by previous studies (e.g., Paux et al., 2005; Andersson-Gunnerås et al., 2006; Liu et al., 2021; Cai et al., 2018; Zinkgraf et al., 2018; Hsieh et al., 2025), such as homologs of *AtFLA12*, or *AtCOBL4*, and poplar TF *PtLBD39*, it also revealed substantial recruitment of processes not previously captured by these studies. Some examples include the existence of dedicated machinery for cellulose and xylan biosynthesis, strong regulation of callose hydrolysis, induction of biosynthesis of rhamnogalacturonan I and its extensive remodeling in the cell wall, and activation of biosynthesis of xylogalacturonan and acidic arabinogalactan II at the onset of G-layer biosynthesis and glucuronoarabinoxylan at the late stage of G-layer formation. We have identified specific members of GT106, GT116, GT92, and GT47 involved in G-layer RG-I biosynthesis and the key candidates for the regulation of the G-layer contractile properties.

High spatial resolution analysis enabled us to pinpoint a set of *XTH* genes activated during TW formation at the time of S-layer deposition rather than during G-layer deposition as previously believed and dedicated clades of *FLA12* genes that are not present in *Arabidopsis* and are induced at the onset of G-layer formation. We also demonstrated that the majority of homogalacturonan-modifying genes and *XTH*s as well as many lignin polymerizing laccases and peroxidases are rapidly suppressed during G-layer biosynthesis, suggesting an active downregulation process. This contrasts with SW-biosynthetic genes, including xylan, cellulose, and monolignol biosynthesis-related genes, which transcripts decrease more slowly.

The dataset also allowed the identification of G-layer-specific genes, including *FLA12-like* arabinogalactan proteins, *PtXTH5*, a LIM actin-associated protein, and transcription factors such as homologs of *SCL18* and *AGL29*. Some of these genes (e.g., *PtAGL6-b* and *FLA12* clades 11A3.1–.4) are apparently found only on some plant species, highlighting their specialized functions. Importantly, the secondary wall master switchers were not involved in G-layer induction, confirming that this layer has a different identity regulated by separate master switchers. Overall, 30 TFs were *de novo*-induced during G-layer formation. These genes likely regulate processes responsible for the structural specialization and contractile properties of G-fibers, offering high-value targets for functional studies. The enrichment of previously uncharacterized GT and GH families additionally suggests that G-layer assembly relies on novel or highly specialized polysaccharide modifications that remain to be elucidated. Moreover, the identification of G-layer-specific biosynthetic modules opens opportunities for biotechnological engineering of cellulose content, fiber mechanics, and biomass quality in forest trees. However, since transcriptomic association alone does not demonstrate causality, future functional validation should be carried out for the identified candidate genes, for example, reverse genetics, CRISPR/Cas editing, tissue-specific overexpression or silencing, and spatial expression analyses.

Beyond providing a foundational reference for *Populus* wood biology, this work establishes a framework for comparative analyses across species forming reaction wood and sets the stage for integrative studies combining transcriptomics with proteomics, metabolomics, cell wall ultrastructure, and mechanical modeling. It complements earlier transcriptomics studies on normal wood development and associated AspWood and NorWood databases (Sundell et al., 2017; Jokipii-Lukkari et al., 2017). As such, this resource not only clarifies long-standing questions about gelatinous fiber differentiation but also provides actionable molecular tools for future research and industrial applications.

## 4 Experimental procedures

### 4.1 Plant material

Four different trees, ca. 4 m high with uniform upright growth, were identified from a natural population of *P. tremula* trees situated in Västerbotten region in Sweden during the summer of 2005. TW was induced in two trees (t08 and t11) by fixing the stem at a 30° angle during the early part of the growing season, while the control two trees (n05 and n08) were allowed to grow in the upright position. On the day of sampling, the trees were felled and the main stem was divided up into approximately 30-cm-long pieces, which were immediately stored on dry ice and kept frozen at −80 °C thereafter until use.

Tangential cryo-sections (30 µm thick) along the developmental gradient across the stem were obtained as described in Uggla and Sundberg (2002). Transverse sections were collected at regular intervals and used to visualize the position of the tangential sections being obtained. Tangential sectioning through the developing wood continued to a depth of up to 1,000 µm. After the sectioning was complete, 18 different pools of the 30-µm sections, which served to prepare RNA libraries, were made according to **Supplementary Table S1**.

### 4.2 Cell wall thickness measurements

Transverse sections of frozen wood blocks previously used for tangential cryo-sectioning and transcript analysis were prepared using a cryo-microtome. They were dehydrated in a graded ethanol series, critical point-dried, mounted onto aluminum stubs with conductive carbon adhesive, and sputter-coated with Gold/Palladium. Imaging was conducted using a scanning electron microscope (FEI Quanta 200, FEI Company, Eindhoven, The Netherlands) operated in low-vacuum mode at a chamber pressure of 0.8 Torr and an accelerating voltage of 20.00 kV using a solid-state backscattered electron detector. Micrographs were acquired at a working distance of 10.7 mm and a magnification of ×503.

Multiple overlapping micrographs were captured across the radial developmental gradient and assembled into a composite image. Cell wall thickness was measured in 10 radial files for each tree: five originating from the apical and five from the basal surfaces of the wood block. The radial distance was calibrated from a common “reference point” shared by both the SEM and transcriptomic datasets and defined as the developmental stage where cell walls maintained structural integrity without deforming during sectioning, corresponding to the initiation of S-layer formation and lignification as confirmed by phloroglucinol-HCl staining. The resulting data points (radial distance vs. cell wall thickness) served to model the developmental trends (**Figure 1**) using polynomial regression.

### 4.3 Sample preparation and RNA extraction

Total RNA was extracted from samples (section pools) with Bio-Rad Aurum Plant Mini Kit and processed through two rounds of amplification with Ambion MessageAmp II aRNA Amplification Kit (Ambion Inc., Austin, Cat#1751). Library generation and paired-end sequencing using Illumina HiSeq 2000 was performed by SciLifeLab (Science for Life Laboratory, Stockholm, Sweden).

### 4.4 RNA-seq processing and expression analysis

RNA-seq data analysis was performed as described in Sundell et al. (2017). Briefly, raw sequencing data quality was assessed via FastQC (version 0.10.1). Sequences were filtered by SortMeRNA (v2.1) and Trimmomatic (v0.32) to detect and remove information from rRNAs and adapters, respectively. Quality control of the sequence data was finalized by an additional run through FastQC. Filtered reads were aligned to the *P. trichocarpa* genome (v3.0 retrieved from Phytozome) using the STAR software (v2-3.0e_r291). Read counting was performed with *HTSeq* (Anders et al., 2015) for each uniquely mapped read to obtain gene-based read counts, followed by read count normalization using variance stabilizing transformation in *DESeq2*, which returned normalized log-transformed counts. Expressed genes were filtered according to two thresholds: first an expression in at least 5 out of 18 libraries and second by having a mean gene expression of at least 1.504, resulting in a total of 22,363 genes after filtering. Expression values corresponded to DESeq2 variance-stabilized (VST, log-transformed) counts. For each gene, we calculated the mean VST value across all samples (row mean of the VST matrix) and kept genes with mean VST > 1.504. This empirically chosen cutoff, based on the distribution of mean VST values, removed the long tail of very lowly expressed genes that primarily contribute noise, while retaining a robustly expressed gene set for co-expression analysis.

The expressed genes were used for PCA. The PCA showed a clear separation between normal and tension wood samples (presented by component 1, which accounts for 40% of the sample variation) but also indicated a shift of the tension wood samples between both replicate trees. Based on this result, all 18 samples were categorized into the following 10 groups corresponding to developmental stages: N1 (n5.1+n8.1), N2 (n5.2+n8.2), N3 (n5.3+n8.3), T1 (t8.1), T2 (t8.2+t11.1), T3 (t8.3+t11.2), T4 (t8.4+t11.3), T5 (t8.5+t11.4), T6 (t8.6+t11.5), and T7 (t11.6). Differentially expressed genes (DEGs) identified with *DESeq2* for different comparisons (N2 vs. N1, N3 vs. N1, T3 vs. mean T1–T2, mean T4–T7 vs. mean T1–T2) were identified based on two criteria: absolute log2 fold change ≥ 1 and pAdj ≤ 0.01. These were plotted in Venn diagram and listed in **Supplementary Tables S3**–**S5**. The expression of all DEGs was shown in a heat map created with heatmap.2 from the R package *gplots*. Hierarchical clustering was done using Euclidean distance and the “ward.D” method, and genes were scaled based on Pearson correlation. Further analyses were done by grouping the 18 samples and using average gene expression. Hierarchical clustering was performed as described above.

Co-expression modules were constructed from all 22,363 expressed genes. For each gene, we used DESeq2 VST-normalized expression values across all developmental stages of normal wood (N1–N3) and tension wood (T1–T7). Expression profiles were z-scaled per gene (standardization to zero mean and unit variance) prior to clustering. Pairwise distances between genes were calculated as Euclidean distances on the scaled expression profiles, and hierarchical clustering was performed using Ward’s minimum variance method. The resulting dendrogram was initially cut into 300 clusters to explore cluster structure. Clusters were then consolidated based on expression coherence and cluster size, yielding 90 co-expression modules that capture major transcriptional programs during wood development. Cluster membership and averaged expression profiles were used for downstream visualization (heat maps and line plots) and for functional enrichment analyses. The code for the analysis is available on GitHub: https://github.com/UPSCb/aspen-tension-wood-development-gradient and the raw data on https://doi.org/10.5281/zenodo.19634726.

Functional enrichment of PFAM domains in different clusters of differentially regulated genes (**Supplementary Table S10**) was carried out by comparing the gene sets in each group to the background of all expressed genes. Fisher’s exact tests were performed for each PFAM domain, and nominal *p*-values were corrected for multiple testing using the Benjamini–Hochberg procedure. Functional enrichment analysis for 90 co-expression clusters (**Supplementary Table S16**-**S18**) was conducted in R using the clusterProfiler package (Yu et al., 2012) within the Bioconductor framework. Terms (MapMan bins, GO terms, and PFAM domains) were parsed and standardized using dplyr, tidyr, and stringr. A term-to-gene mapping (TERM2GENE) was constructed, and the enrichment was tested against a custom background universe comprising all expressed genes. Overrepresentation analysis (ORA) was performed using enricher() from clusterProfiler, which applies the hypergeometric test (equivalent to Fisher’s exact test) to assess whether the overlap between the group proteins and the annotation terms exceeds random expectation. Adjusted *p*-values were calculated using the Benjamini–Hochberg procedure.

## Data Availability

The datasets presented in this study can be found in online repositories (**Supplementary Material**). The name and address of the repository for the raw RNA data can be found in section 4 of the article.

## Supplementary material

Supplementary Figure 1 Phytozome wide analysis of fasciclin arabinogalactan proteins (FLAs).

Supplementary Figure 2 Expression of unknown function glycosyl transferase- and glycoside hydrolase- encoding genes showing upregulation during G-layer biosynthesis.

Supplementary Figure 3 Gene co-expression clusters based on variance-stabilized expression patterns in normal wood (NW, trees n05 and n08) and tension wood (TW, trees t08 and t11) developmental series.

Supplementary Table 1 Localization of samples used for RNA analysis.

Supplementary Table 2 List of all expressed genes in all samples, classified according to the common expression patterns into 90 clusters.

Supplementary Table 3 Differentially-regulated (pAdj ≤ 0.01; |log2 fold change ≥ 1|) genes (DRG) at the early stage of G-layer formation, compared to the stage prior to G-layer formation in developing tension wood (T3 vs mean T1-T2).

Supplementary Table 4 Differentially-regulated (pAdj ≤ 0.01; |log2 fold change| ≥ 1) genes (DRG) at the late stage of G-layer formation, compared to the stage prior to G-layer formation in developing tension wood (mean T4-T7 vs mean T1-T2).

Supplementary Table 5 Differentially-regulated (pAdj ≤ 0.01; |log2 fold change| ≥ 1) genes (DRG) during normal wood differentiation, compared to the initial stage (N1).

Supplementary Table 6 Differentially regulated genes as shown in the Venn diagram in **Figure 3A**.

Supplementary Table 7 Differentially expressed genes during early stages of G-layer differentiation compared to the stage prior G-layer deposition and their VST expression values in individual samples collected across different wood developmental zones.

Supplementary Table 8 Differentially expressed genes during all stages of G-layer differentiation compared to the stage prior G-layer deposition and their VST expression values in individual samples collected across different wood developmental zones.

Supplementary Table 9 Differentially expressed genes during late stages of G-layer differentiation compared to the stage prior G-layer deposition and their VST expression values in individual samples collected across different wood developmental zones.

Supplementary Table 10 PFAM enrichment in different clusters of differentially expressed genes shown in **Figure 3E**.

Supplementary Table 11 All expressed CAZYme genes sorted according to their function in cell wall biosynthesis (Kumar et al., 2019).

Supplementary Table 12 All Identified fasciclin-like AGPs (FLAs) of clade 11A as shown in **Supplementary Figure S1**.

Supplementary Table 13 Phylogeny of fasciclin-like AGPs (FLAs) of clade 11A as shown in **Supplementary Figure S1**.

Supplementary Table 14 Callose hydrolase genes of GH17, their domain structures and expression in TW.

Supplementary Table 15 All expressed nucleotide sugar transporter genes.

Supplementary Table 16 All expressed monolignol biosynthetic genes, laccases and peroxidases.

Supplementary Table 17 List of all enriched Mapmann functional categories for each co-expression cluster sorted according to their significance within each cluster.

Supplementary Table 18 List of all enriched GO terms for each co-expression cluster sorted according to their significance within each cluster.

Supplementary Table 19 List of all enriched PFAM terms for each co-expression cluster sorted according to their significance within each cluster.

Supplementary Table 20 Groups of co-expression clusters sharing similar expression pattern.

Supplementary Table 21 *De-novo*-induced genes during G-layer formation.

Supplementary Table 22 Genes related to hormones with GL1 and GL2 patterns of expression.

Supplementary Table 23 Transcription factors and their VST expression in normal wood (N1-N3) and tension wood (T1-T7).
