# Supplementary material for: A high spatial resolution transcriptomics of tension wood reveals the genetic program of contractile G-layer formation
Source: Front Plant Sci. 2026 Sep 23;17:1929884. doi: 10.3389/fpls.2026.1929884 (PMC13642294; doi:10.3389/fpls.2026.1929884)
Supplement: Supplementary file 1 [file DataSheet2.docx]

Supplementary Material

A high spatial resolution transcriptomic of tension wood reveals genetic program of contractile G-layer formation

Manoj Kumar^1€*^, Carolin Grones^1¤^, Holly Allen^2^, Judith Lundberg-Felten^1^, Nicolas Delhomme^1^, Bjorn Sundberg^1^, Ewa J. Mellerowicz^1*^

*** Correspondence:**
Ewa J. Mellerowicz

ewa.mellerowicz@slu.se

Manoj Kumar

manoj.kumar@manchester.ac.uk

# 1 Supplementary Figures

# Short legends for Supplementary Figures

**Figure S1.** Phytozome wide analysis of fasciclin arabinogalactan proteins (FLAs).

**Figure S2.** Expression of unknown function glycosyl transferase- and glycoside hydrolase- encoding genes showing upregulation during G-layer biosynthesis.

**Figure S3.** Gene co-expression clusters based on variance-stabilized expression patterns in normal wood (NW, trees n05 and n08) and tension wood (TW, trees t08 and t11) developmental series.

**Supplementary Figure 1.** **Phytozome wide analysis of fasciclin arabinogalactan proteins (FLAs).** **A.** An overview. Previously identified FLAs (AtFLA01-21 and PtFLA12s) were used as query in BLAST searches at Phytozome (V14.2). Additionally same sequences were used as query at PopGenie to retrieve POTRA and POTRS sequences. After removing duplicate hits and multiple splice variants, 1199 proteins retained and classified into C11A-E within CLANS. **B.** Phylogenetic tree of CLANS cluster C11A. Protein sequences were aligned with MUSCLE and a phylogenetic tree was produced using FASTREE. Visualisation and colouring was performed with Archaeopteryx.

**Supplementary Figure 2. Expression of unknown function glycosyl transferase- and glycoside hydrolase-encoding genes showing upregulation during G-laye biosynthesis.** Average VST expression. Stars indicate differentially regulated genes during early and/or late phase of G-layer synthesis.

**Supplementary Figure 3. Gene co-expression clusters based on variance-stabilized expression patters in normal wood (NW, trees n05 and n08) and tension wood (TW, trees t08 and t11) developmental series.** Data are means of all genes within the cluster ± SE
